# Epidemiological Trends of Dengue Disease in Thailand (2000–2011): A Systematic Literature Review

**DOI:** 10.1371/journal.pntd.0003241

**Published:** 2014-11-06

**Authors:** Kriengsak Limkittikul, Jeremy Brett, Maïna L'Azou

**Affiliations:** 1 Faculty of Tropical Medicine, Mahidol University, Bangkok, Thailand; 2 sanofi-aventis Singapore Pte Ltd, Singapore; 3 Global Epidemiology Department, Sanofi Pasteur, France; Pediatric Dengue Vaccine Initiative, United States of America

## Abstract

A literature survey and analysis was conducted to describe the epidemiology of dengue disease in Thailand reported between 2000 and 2011. The literature search identified 610 relevant sources, 40 of which fulfilled the inclusion criteria defined in the review protocol. Peaks in the number of cases occurred during the review period in 2001, 2002, 2008 and 2010. A shift in age group predominance towards older ages continued through the review period. Disease incidence and deaths remained highest in children aged ≤15 years and case fatality rates were highest in young children. Heterogeneous geographical patterns were observed with higher incidence rates reported in the Southern region and serotype distribution varied in time and place. Gaps identified in epidemiological knowledge regarding dengue disease in Thailand provide several avenues for future research, in particular studies of seroprevalence.

**Protocol registration:**

PROSPERO CRD42012002170

## Author Summary

We conducted this comprehensive systematic review to determine the impact of dengue disease in Thailand for the period 2000–2011, and to identify future research priorities. Well-defined methods were used to search and identify relevant published research, according to predetermined inclusion criteria. In addition to information from studies published in the literature, the review draws largely on surveillance data from the Annual Epidemiological Surveillance Reports published by the Thailand Ministry of Public Health. The pattern of annual number of reported dengue cases over the review period was complicated by epidemic years; consequently, a trend in the number of reported cases could not be identified. It was apparent that despite a shift in age group distribution dengue from younger towards older persons, dengue in Thailand remains a predominantly childhood disease. The seasonality and heterogeneous spatial and temporal nature of the disease were confirmed. It is clear that the nationwide passive surveillance system is a source of consistent data relating to severity, age and serotype. However, several gaps were identified that would benefit the understanding of dengue epidemiology in Thailand, such as seroprevalence data and a record of the proportion of reported cases that are hospitalized.

## Introduction

Dengue is a global arboviral disease affecting humans. The primary vector is the *Aedes aegypti* (Linnaeus) mosquito. Dengue is present in the tropical and subtropical regions of the Americas, the eastern Mediterranean, Africa, and the World Health Organization (WHO) Western Pacific and Southeast Asia^ regions^ [1]. Countries included within regions designated as Southeast Asia differ according to WHO, political and geographic definitions. Unless otherwise stated, the term Southeast Asia used in this paper refers to the WHO Southeast Asia Region (SEAR).

Globally, more than 2.5 billion people are at risk [1]. The WHO estimates that more than 50 million dengue virus (DENV) infections and 20,000 dengue disease-related deaths occur annually worldwide [2], [3], and a recent disease distribution model estimated there were 390 million DENV infections in 2010, including 96 million apparent infections (i.e., cases that manifest at any level of clinical or subclinical severity). Overall, 70% of these apparent infections occurred in Asia [4].

Thailand observed its first cases of dengue disease in 1949; sporadic cases continued to be reported throughout the 1950s [5], [6] and the first major outbreak of dengue haemorrhagic fever (DHF) was reported in Bangkok in 1958 [7], [8]. There were 2158 cases and 300 deaths in this outbreak [9]. DENV infection is caused by any one of four distinct DENV serotypes (DENV-1, -2, -3 or -4) [1]. Three or possibly four virus types (DENV-1, possibly DENV-2 and two unidentified serotypes) were isolated during the 1958 epidemic [10], and the co-circulation of all four dengue serotypes was demonstrated in the early 1960s in Bangkok [11]. By the late 1970s, the disease was widespread among countries in Southeast Asia and DHF had become a leading cause of hospitalization and death among children in Thailand [12]. There was a major epidemic of dengue disease in 1987, in which 174,285 cases were reported, after which the number of reported cases remained relatively low and stable, with under 100,000 cases reported each year. Two large outbreaks were reported in 1997 and 1998, with 101,689 and 126,348 cases reported, respectively [9], [13]. Before 2004, Thailand reported the highest number of annual dengue disease cases in Southeast Asia, with an average of almost 69,000 cases per year reported between 1985 and 1999 [13]. After 2004, Indonesia reported the highest number of cases from the region, accounting for 57% of the cases reported to the WHO Southeast Asia region in 2006 [13]. The epidemiology of dengue disease in Thailand is characterized by cyclical epidemic activity alternating between years of relatively low and high dengue disease incidence [14], [15].

A reporting system for dengue surveillance in Thailand started in 1958. The national surveillance system for DHF was initiated in 1972 by the Bureau of Epidemiology (BoE), Thai Ministry of Public Health (MoPH) becoming fully operational in 1974 [16]. DF was included in the surveillance system in 1994. Reports for patients diagnosed with dengue disease are collected from hospital in-patients and hospital out-patients from health facilities nationwide, all government hospitals and some private hospitals and clinics (the reporting sites are mostly public hospitals, with a few voluntary reports from private hospitals). It is mandatory to report the confirmed cases, but not for the suspected cases (which is subject to the physicians' willingness to report). The reporting form (Form 506) is used to record demographic data — age, sex, day of onset and the address (locality) where the case occurred, categorised as municipalities (‘cities’ or ‘suburbs’) or ‘other’ (mostly rural) areas [16]. It should be noted that all reported dengue disease cases in Thailand are diagnosed by the trained physician using WHO case definition established since the 1970s, which classify dengue into dengue fever (DF), DHF and dengue shock syndrome (DSS) [17]. Digital or hardcopy reports of dengue disease are transmitted up the system from the local level, initially to provincial health offices and then to the BoE where they are collated and analyzed. Prior to 1999 the reports were sent by post; electronic transmission of reports began in 1999 [16]. For the past 10 years, the Thai surveillance system at the central level has been systematic and relies on electronic-based data although at the local level there is no compulsory electronic proforma, indeed hospitals often generate their own software programs that are compatible with Form 506 for entering and transferring data. Epidemiological data on DF, DHF and DSS in Thailand are disseminated from central departments in the form of weekly newsletters (the BoE Weekly Epidemiological Surveillance Report) and published online on the MoPH website within the Annual epidemiological surveillance reports (AESRs).

Dengue disease laboratory diagnostics in Thailand can be ordered on an individual basis and include dengue virus isolation, viral genome detection by reverse transcription polymerase chain reaction (RT-PCR), four-fold increases of paired sera (haemagglutination inhibition) or IgM >40 U or IgG increasing >100 U. Virological surveillance (virus isolation and serotyping by RT-PCR) is performed by the Department of Medical Science, especially before the outbreak season, which appoints a number of hospitals from around the country to act as sentinel sites. However, only a small proportion of reported cases are tested for DENV infection. Furthermore, the proportion of specimens sent for testing varies between provinces in each region.

Thailand is divided politically into 76 provinces, with the capital, Bangkok, being a special administrative area. A four-region administrative system is used by the MoPH (**Figure S1**): North (population 11.5 million), Northeast (18.8 million), Central (including Bangkok) (26.3 million), and South (8.9 million) [18]. Thailand has three types of climate, a tropical rain climate in the coastal areas of the east and south, a tropical monsoon climate in the southwestern and southeastern coastal areas, and a tropical wet and dry or savannah climate in the southwest, central and northern regions. Climatic factors such as temperature, rainfall and relative humidity affect the growth and dispersion of the mosquito vector and are known to be associated with dengue outbreaks [19]. In common with other developing tropical and subtropical countries, Thailand has population demographics and socio-economic conditions that favour dengue transmission, such as rapid population growth and rural–urban migration [20], and densely populated areas that provide suitable *Aedes* mosquito larval habitats [1], [21].

This review describes the epidemiology of dengue disease in Thailand reported in the literature between 2000 and 2011 in the context of the national and regional trends and aims to identify gaps in epidemiological knowledge requiring further research. Incidence (by age and sex), seroprevalence and serotype distribution and other relevant epidemiological data such as geographical distribution are described.

We estimated that a time period of at least 10 years would allow observation of serotype distribution over time and through several epidemics and, in view of the 3–5-year periodicity of dengue outbreaks [7], would also accurately reflect recent changes in dengue disease epidemiology. We set the start date as 1 January 2000, as opposed to an earlier date, to limit the bias that any differences in surveillance practices over time would have on the results. The cut-off for our review period was set as 28 February 2012, the date when we initiated this review.

## Methods

The overall methodology, search strategy, and inclusion and exclusion criteria for this literature analysis and review are included in a protocol that was developed by a Literature Review Group (LRG). The protocol was based on the preferred reporting items of systematic reviews and meta-analyses (PRISMA) guidelines [22]. The protocol was registered on PROSPERO, an international database of prospectively registered systematic reviews in health and social care managed by the Centre for Reviews and Dissemination, University of York (CRD42012002170: http://www.crd.york.ac.uk/PROSPERO/display_record.asp?ID=CRD42012002170) on 23 March 2012.

### Search strategy and selection criteria

The LRG guided the literature analysis process, defined the search strategy, and prepared the protocol and review documents. Specific search strings for each database were designed with reference to the expanded Medical Subject Headings thesaurus, encompassing the terms ‘dengue’, ‘epidemiology’, and ‘Thailand’. Different search string combinations were used for each electronic database with the aim of increasing the query's sensitivity and specificity. Searches of selected online databases (**Table S1**) were conducted between 9 February 2012 and 28 February 2012.

As stated in the protocol, studies (as well as conference materials, grey literature and official reports and bulletins) published in either Thai or English between 1 January 2000 and 28 February 2012 were included in the analysis. References not meeting these criteria that were found in databases that did not allow language and/or date limitations were deleted manually at the first review stage. No limits by sex, age and ethnicity of study participants or by study type were imposed, although single-case reports and studies that only reported data for the period before 1 January 2000 were excluded, as were publications of duplicate data sets, unless the articles were reporting different outcome measures. Editorials and reviews of previously published data were also excluded. Additional publications not identified by the search strategy, unpublished reports and grey literature were included if they met the inclusion criteria and were recommended by the LRG.

Sources were reviewed by the LRG to ensure they complied with the search inclusion and exclusion criteria. Following a review of the source titles and abstracts, during which duplicates were removed, the LRG performed a second review of the full text of any published sources selected to make the final selection of relevant sources to include. In an amendment to the original protocol the Literature Review Group sanctioned the extraction of surveillance data for 2011 from the MoPH Bureau of Epidemiology Surveillance Database website on 16 July 2012.

We chose not to exclude articles and other data sources nor formally rank them on the basis of the quality of evidence. Whilst an assessment of study quality may add value to a literature review, we were of the view that given the expected high proportion of surveillance data among the available data sources and the nature of surveillance data (passive reporting of clinically-suspected dengue), such quality assessment would not add value to our review.

The selected data sources were collated and summarized using a data extraction instrument developed as a series of Excel (Microsoft Corp., Redmond, WA) spreadsheets. Data from literature reviews of previously published peer-reviewed studies and pre-2000 data published within the search period were not extracted. The original data sources and the extraction tables were made available to all members of the LRG for review and analysis. In view of the expected heterogeneity of eligible studies in terms of selection and number and classification of cases, a meta-analysis was not conducted; a narrative synthesis of our findings is presented. For the purposes of the analysis, we defined national epidemics as those years in which the number of cases was above the 75^th^ percentile for the period.

## Results

### Literature survey and analysis

**Figure 1:**
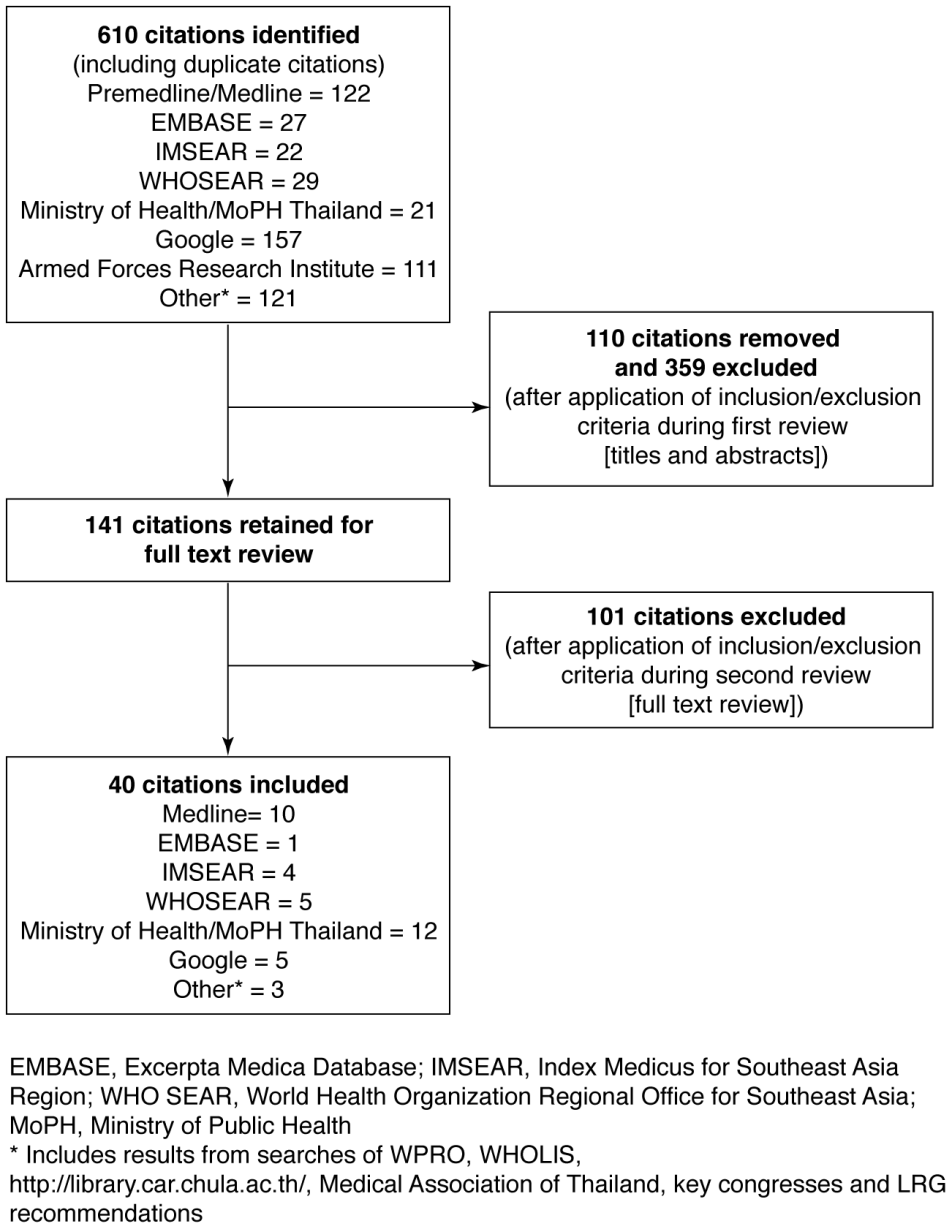
Results of literature search and evaluation of identified data sources according PRISMA. Duplicates and articles that did not satisfy the inclusion criteria were removed following evaluation of the titles and abstracts. The full text of these documents was examined to facilitate the final selection of relevant articles. Included publications were collated and summarized using a data extraction instrument developed as a series of spreadsheets, as described in the protocol.

This review concentrates on national epidemiological data collated from several sources, including the latest data from the Thailand MoPH (Table S1). The literature searches identified 610 relevant data sources; of these, 40 fulfilled the inclusion criteria for the analysis (**Figure 1** **; Table S2**). Most national epidemiological data were derived from the annual surveys or statistical tables produced by the MoPH (12 sources [23]–[34]). Of the remaining 28 articles and reports, the majority were journal articles that mainly described regional epidemiological data derived from surveys and studies conducted in individual regions and provinces (**Table S2**) and are used to support the national data with regard to regional incidence, serotype and age distribution.

### National epidemiology

The AESRs published by the MoPH provide a source of country-wide reporting of dengue disease statistics for 2000–2011 [23]–[34], albeit with some missing data owing to reporting variations.

**Figure 2:**
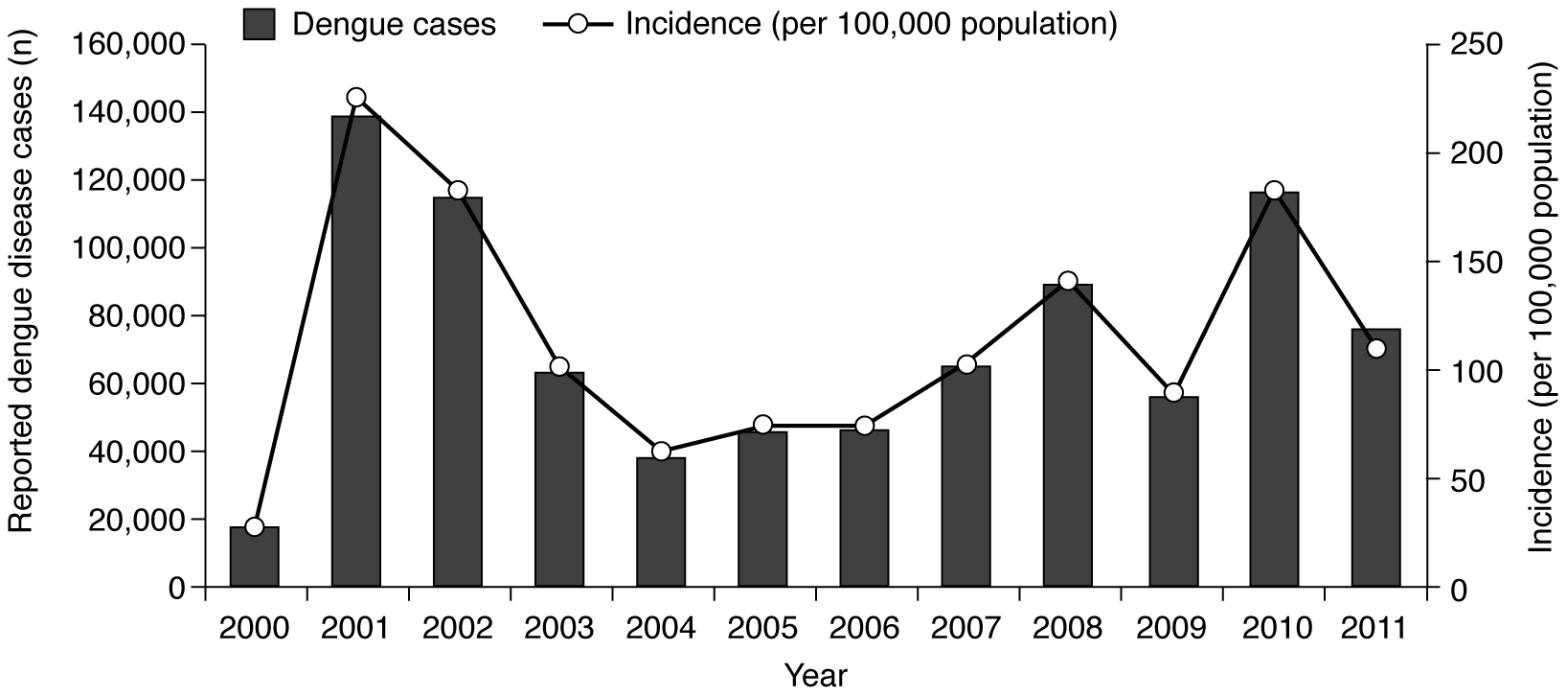
Number of reported dengue disease cases and dengue disease incidence, Thailand, 2000–2011 [23]–[34]. The number of reported dengue disease cases is plotted against the left-hand vertical axis. Peaks in the number of cases (national epidemics) that were above the 75^th^ percentile for the period occurred in 2001, 2002 and 2010; another peak (which was at the 70^th^ percentile) occurred in 2008. The incidence of dengue disease per 100,000 population is plotted against the right-hand vertical axis. The incidence averaged 115 cases annually between 2000 and 2011. No clear trend over time in the number or incidence of reported dengue disease cases could be discerned as the pattern over the review period was complicated by epidemic years.

Between 2000 and 2011, more than 860,000 dengue disease cases were reported, corresponding to an annual average of approximately 72,000 cases and 100 deaths, and an average annual incidence of 115 cases per 100,000 population. Peaks in the number of cases (national epidemics) that were above the 75^th^ percentile (102,213) for the period occurred in 2001, 2002 and 2010, when 139,355 cases (incidence rate 224/100,000 population), 114,800 cases (183/100,000), and 116,947 cases (177/100,000) were reported, respectively; another peak of 89,626 cases (142/100,000), which was at the 70^th^ percentile, occurred in 2008 (**Figure 2**). The lowest incidence occurred in 2000 (30.14/100,000) [23]–[34].

**Figure 3:**
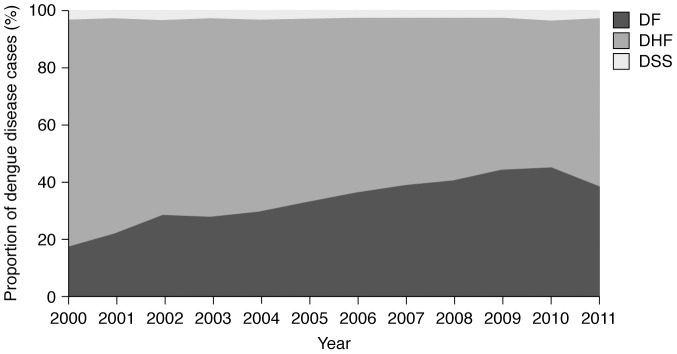
Reported cases of dengue fever, dengue haemorrhagic fever and dengue shock syndrome, Thailand, 2000–2011 [23]–[34]. The contribution of DSS to the total number of dengue disease cases remained relatively stable over the decade. Over the same period, the proportion of the total dengue disease cases classified as DF tended to increase year-on-year (with the exception of 2010–2011) whereas the contribution of DHF to the total number of dengue disease cases decreased. DF, dengue fever; DHF, dengue haemorrhagic fever; DSS, dengue shock syndrome.

Since 2002, the proportion of the total number of reported cases of DF and DHF reversed, while the proportion of DSS remained relatively stable over the decade, ranging between approximately 1% and 3% (**Figure 3**) [23]–[34]. The reasons for the change in DF∶DHF ratio change are more probably more related to reporting behaviours than changes to the reporting system. During the 2000–2011 period there were no fundamental changes to the case reporting system. There has however been a change in reporting behaviour over the review period. A DF diagnosis relies on voluntary reports, which reflect to physicians' attention and willingness to report and their sense of importance of this matter. Moreover, in 1999 the King's Project (a large prevention and control programme for dengue) was introduced in which the aim was to increase people's knowledge of the disease through education and television advertisements [35]. Consequently, patients attended hospital earlier resulting in early diagnosis. Physicians cooperated with the programme by reporting DF, whereas previously reporting was focused on severe forms of dengue (DHF and DSS). Improved physician awareness to the disease was assisted by better diagnostic capabilities. In particular, laboratory facilities improved in many areas and the results were reported back to local hospital from the central laboratory faster than before. In addition, following the 2005 avian flu outbreak more PCR laboratories became available at the regional level [26]. Greater diagnostic capabilities (possibly through the wider use of immunoglobulin G (IgG)/IgM test kits and the NS1 antigen test, which may help confirm the diagnosis of mild dengue virus infections) and changes in surveillance methods may also have contributed to the increasing proportion of DF cases detailed in the AESR in recent years.

The number of deaths due to dengue disease reported between 2000 and 2011, and the mortality rate (deaths per 100,000 population), broadly reflect the number and incidence of cases reported. There were 1216 deaths reported from dengue disease between 2000 and 2011, an average of 0.16 deaths per 100,000 population [23]–[34]. The highest mortality rate occurred during the large 2001 epidemic (245 deaths, 0.39 deaths/100,000 population) [23], [24]. Between 2003 and 2011, the average case fatality rate (CFR) reported by the MoPH for DHF was 0.05% (0.03–0.09) and for DSS it was 4.45% (range: 4.04–5.92); the highest CFR for DSS was in 2006 (5.92%) [26]–[34]. There were no deaths attributed to DF over this period.

No clear trend over time in the number of reported dengue disease cases could be discerned as the pattern of the annual number of cases of dengue disease over the review period was complicated by epidemic years. There was an overall decline in case fatality rates reported between 2000 and 2010, reflecting rates reported in most of the dengue disease endemic countries in the Southeast Asia region between 1995 and 2000 [36]. These patterns may be the result of changes in reporting and improvements in case management.

### Regional epidemiology

**Figure 4:**
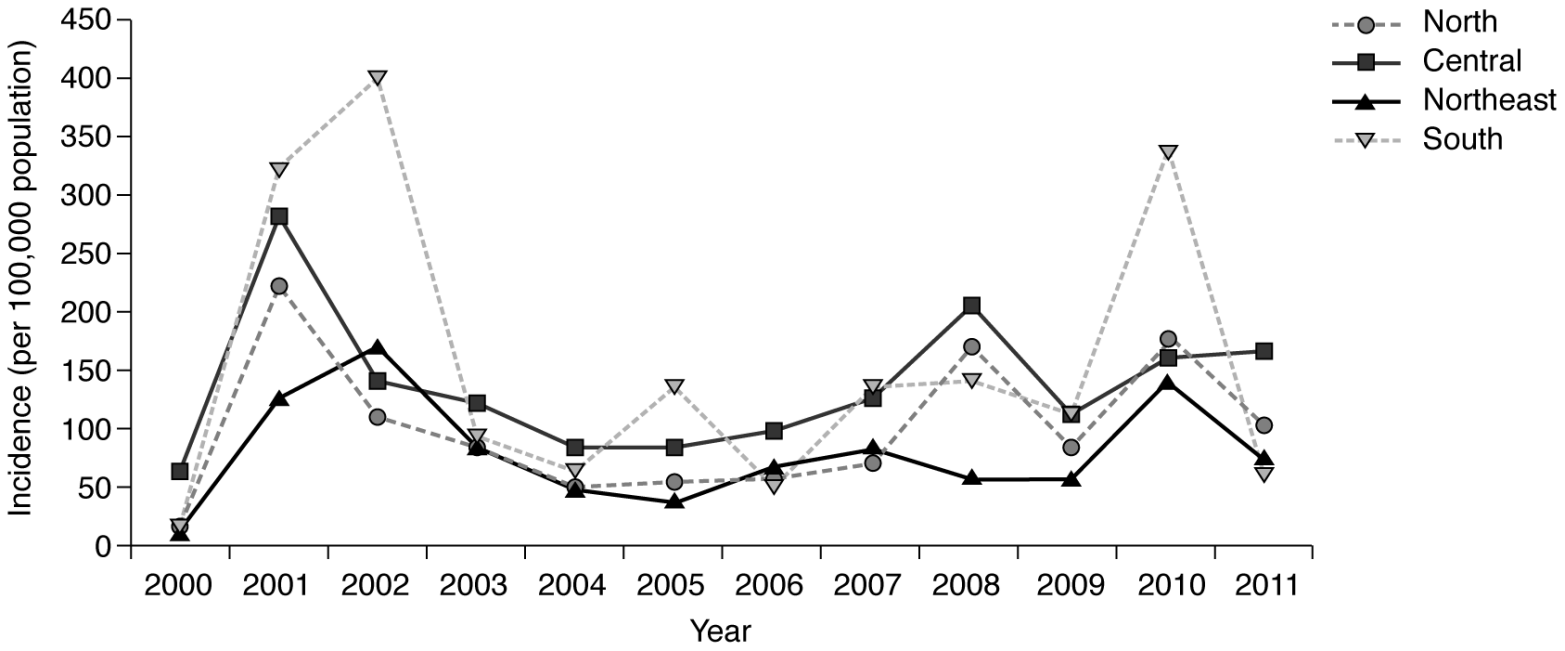
Reported dengue disease incidence by region, Thailand, 2000–2011 [23]–[34]. The patterns of regional dengue disease case numbers broadly reflected those observed nationally. The reported incidence of dengue disease was highest in the South region in 2001, 2002, 2005, 2007 and 2010.

Annual dengue disease case numbers evident at the national level were broadly repeated at the regional level. Consistent with its higher population density, the Central region reported the highest number of dengue cases in most years and the most deaths over the period of the review. The most cases reported was in 2002 from the northeast region (37,191 cases) [23]–[34]. The reported incidence rate was highest in the southern region in 2001, 2002, 2005, 2007 and 2010 (**Figure 4**, Table S3); the incidence rate in the southern region in 2002 was more than double that reported in the other regions (402.54/100,000 population) [23]–[34]. The highest mortality rate was reported from the southern region in 2001 and 2002 (0.77/100,000 population), as well as in 2010 (0.66/100,000 population) [23]–[34].

Available regional data on the proportion of DF, DHF and DSS cases for the years 2003–2011 [26]–[34] show similar increases in the proportion of reported DF cases to those seen at the national level (Central: from 20% to 39%; North: from 33% to 46%; Northeast: from 31% to 46%; South: from 34% to 48%).

### Age and sex distribution of dengue disease

**Figure 5:**
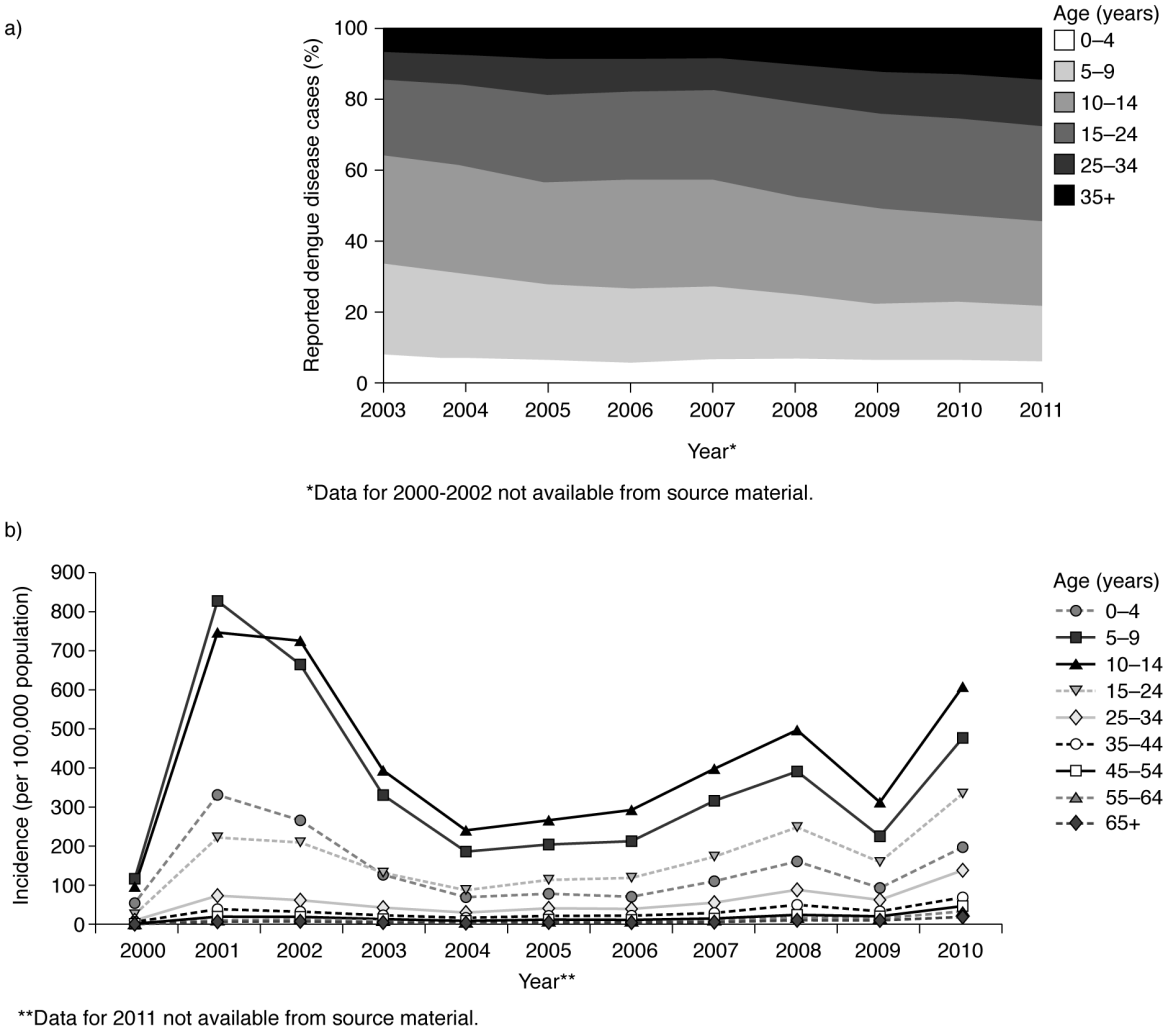
Pattern of reported cases and incidence of dengue disease, by age, Thailand, 2000–2011 [23]–[34]. Dengue disease is a disease of children and young adults in Thailand. Panel (a) shows that most reported cases between 2003 and 2011* are in individuals aged between 5 and 24 years. There was a shift in age group predominance of dengue disease over the survey period from younger towards older individuals. The age-related incidence per 100,000 population between 2000 and 2010 (b) reveals the highest incidence rates were in those aged 10–14 years. *Age bands for 2011 differ (0–5, 6–10, 11–14, 15–24, 25–34, 35–44, 45–54, 55+) and do not match bands for disease numbers: consequently, some 2011 incidence figures are estimates.

In Thailand, dengue remains a disease of children and young adults, with most cases occurring in individuals aged between 5 years and 24 years, who represent one third of the population (Table S4). However, the age group with the highest incidence changed from those aged 5–9 years to those aged 10–14 years in 2002, and there has been a general shift in age group predominance of dengue disease over the survey period from younger towards older individuals over 15 years of age [31]–[34], [37]–[39] (**Figure 5a and 5b**), continuing a trend that was first observed in the 1980s [36], . These findings are consistent with a recent publication reporting a significant increase in the age at dengue exposure in December 2010 in Rayong Province, Southeast Thailand [42].

Throughout the review period, relatively more cases of severe dengue (DHF and DSS) were reported in individuals aged between 5 and 14 years compared with those aged 15 years or older. In particular, DSS was less common in individuals aged 15 years or older. Data from cohort studies indicate that many children in the 5–14 years age group may be experiencing a second infection of dengue, which could account for the high incidence of severe disease in this age group [41]. Lower incidence rates of severe disease in older age groups could be due to reduced exposure to infection or reduced severity of disease in individuals experiencing their third or fourth infection [43].

Over the review period, approximately 70% of deaths due to dengue disease reported to the MoPH were in patients younger than 15 years. Typically, the highest CFRs were seen either in young children aged 0–4 years or in older adults aged 55–64 years, a trend that likely reflects the susceptibility of the young and old to more adverse consequences of dengue disease and its clinical management, as well as the risk associated with comorbidities in older adults [44]. However, the number of reported cases in those aged 55 years and above is small compared to the other age groups. Individuals aged over 65 years had the lowest reported incidence rate of dengue overall, and the only case fatalities in this age group were recorded in 2001, 2005 and 2010.

Comparable regional data for age-related distribution of dengue were not recorded in the studies selected for this review. Individual studies suggest a pattern similar to that seen nationally, with younger age groups more likely to contract dengue than adults and the elderly [37]–[39].

Although more females than males were reported to have the disease in 2009 (male∶female ratio 1∶1.6) [32], [45], [46], in general, slightly more males than females were affected by dengue over the survey period, with reported male∶female ratios of between 1.1∶1 and 1.2∶1 [26], [29]–[31], [33]. These differences may be due to differences between the sexes in health-seeking behaviours in Thailand [37].

### Seasonal factors

**Figure 6:**
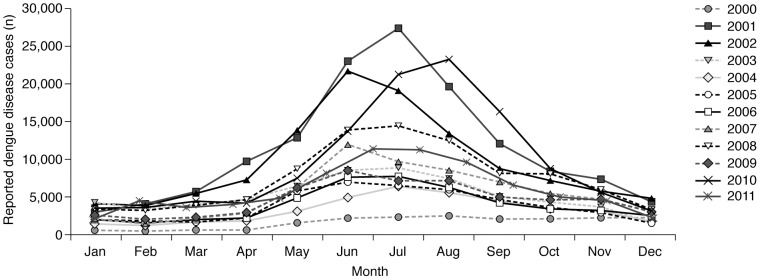
Number of reported cases due to dengue disease, by month, Thailand, 2000–2012 [23]–[34]. Most of Thailand has a tropical wet and dry or savannah climate, where the wettest months are usually August–October. Country-wide, average annual temperatures range from 19°C to 38°C, with higher temperatures in the dry season (November to March). The available data show a seasonal peak in the numbers of cases due to dengue between May and September reflecting seasonal changes in climate and the association between the wettest months and vector activity.

The available data show a seasonal peak in the numbers of cases (**Figure 6**) and deaths (data not shown) due to dengue between May and September annually [23]–[34], [47], [48] which is probably due to seasonal changes in climate [14], and the association between the active season of the vectors and the wettest months. Thus the pattern coincides with the rainy season in Thailand, which, although it varies slightly from region to region and is largely dominated by the monsoon, can be classified broadly as May/June to October.

### DENV serotype distribution

**Figure 7:**
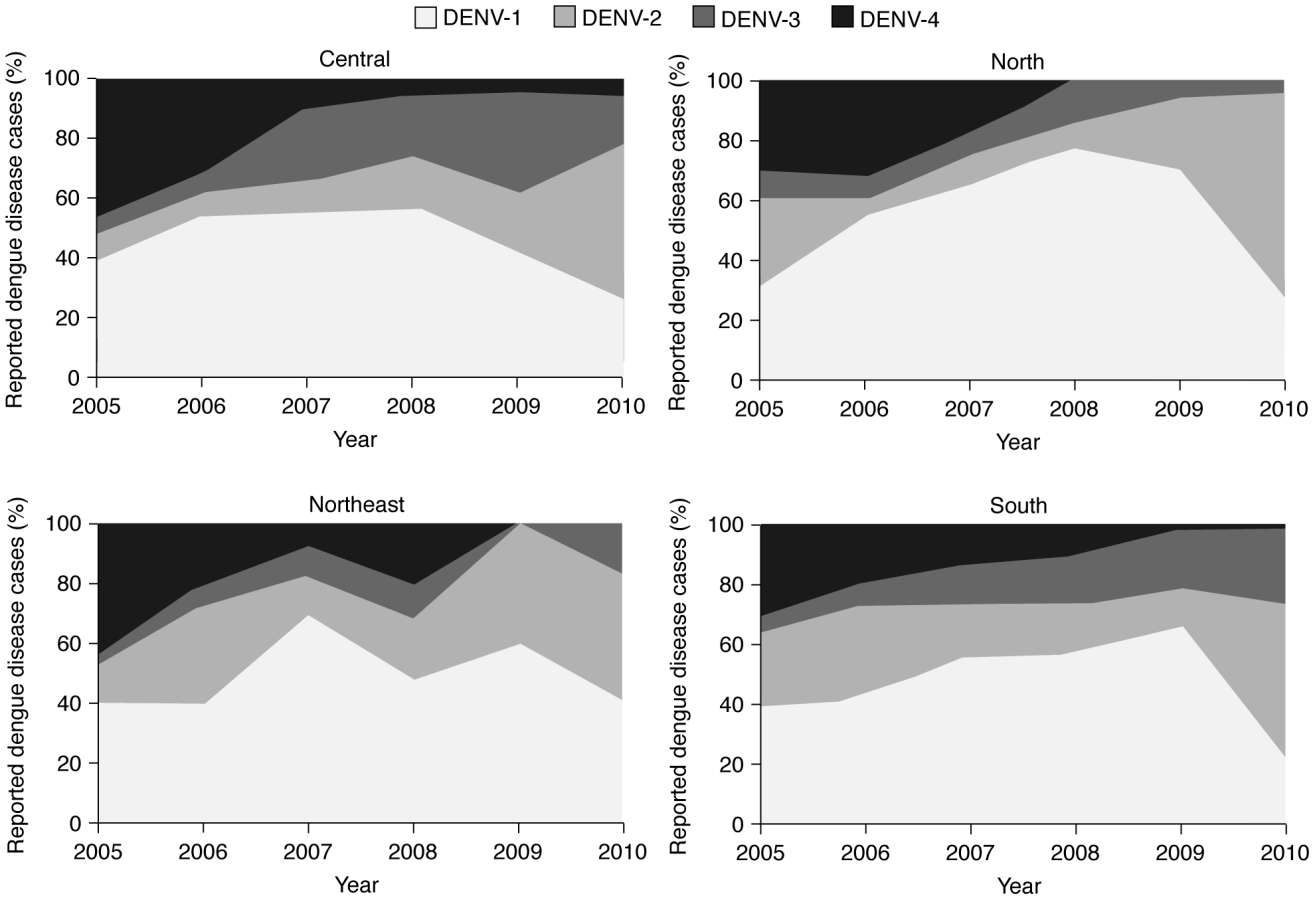
Change in pattern of circulating dengue virus serotypes by year and region, Thailand, 2005–2010 [28]–[33]. Regional DENV serotype data for the period 2005–2010 show similar patterns in each region. Broadly, there was a reduction in the proportion of DENV-1 and an increase in the proportion of DENV-2 in all regions. The proportion of DENV-3 was variable by time and between regions, whereas DENV-4 only remained in circulation throughout this 5-year period in the Central region. DENV, dengue virus. *Data for 2000–2004 not available from source material.

At the time of this review, comprehensive regional DENV serotype data for Thailand from the MoPH AESRs were only available for the period 2005–2010. These serotype data show a broadly similar pattern in each region, with a reduction in the proportion of DENV-1 and an increase in the proportion of DENV-2 isolates over that period (**Figure 7**) [28]–[33]. DENV-4 peaked during 2005 and 2006 and then declined, but remained in circulation in the Central region throughout this 5-year period, albeit at a decreasing percentage of all dengue disease cases (4.6–6.2% during 2008–2010, compared with 10.4% in 2007 and 46.1% in 2005). By contrast, in 2009 and 2010, DENV-4 was not isolated in samples from the North or Northeast regions and was reported at <2% in the South region. DENV-3 circulated in all regions throughout the whole period.

In general, between 2000 and 2010, DENV-1 and DENV-2 were the most commonly reported serotypes in national and/or regional studies in Thailand [23]–[33], [39], [48]–[53] (Table S5). However, during the years 2000–2002 and 2008–2010, DENV-3 was more commonly identified than during the middle part of the decade [23]–[33]; between 2003 and 2008, reports of DENV-4 were more common than during 2000–2002 and 2009–2010. In 2010, the most commonly identified serotype was DENV-2, representing over half of all those isolated (54.6%), followed by DENV-1 (25.5%), DENV-3 (15.3%) and DENV-4 (4.6%) [33]. Anantapreecha et al. also found similar temporal and spatial changes in the predominant DENV serotype [50], [54], [55].

### Apparent and inapparent infections

Whereas most epidemiological reports of dengue in Thailand address the magnitude of clinically apparent infections, a number of studies published during the review period investigated both apparent and inapparent infections. Inapparent infections may have important public health implications in understanding virus transmission and the pathogenesis of dengue disease illness [56].

A variable proportion of inapparent infections relative to clinically apparent infections were reported [48], [56]–[60]. An active case surveillance in 2119 primary school children (median age 9.3 years) in a rural setting in Kamphaeng Phet, North region, by Endy et al. reported an overall incidence of dengue infection for the year 2000 of 2.2%: 0.8% symptomatic infections and 1.4% clinically inapparent infections, a symptomatic to inapparent ratio (S∶I ratio) of 1∶1.75 [48]. A later study reported an overall S∶I ratio of 1∶3 for the period 2004–2008 [56], [57]. Similarly, during a large DF/DHF disease outbreak in Nakhon Pathom province (Central region) in 2001, 8.8% of individuals (age range: 0 years to over 50 years) had an inapparent infection with dengue virus, as determined by IgM positivity, over a 2-month period between March and April. Most of the serologically positive individuals (80.8%) reported no previous fever [58].

Like many surveillance programmes the starting point for reporting in Thailand is a visit to a healthcare provider or hospitalization. As such, the national surveillance data may be incomplete and likely under-reported similar to other Southeast Asian countries [61]. A recent analysis of data from prospectively followed cohorts with laboratory confirmation of dengue cases show that dengue incidence is under-recognized in Thailand and Cambodia by more than eight-fold [62]. Consequently changes in the level of healthcare attendance or in the level of reporting to surveillance system by physicians (as discussed above) may also affect the under- or over-reporting of dengue disease.

### Evidence gaps

Epidemiological knowledge in Thailand benefits from a nationwide surveillance system including virological surveillance, complemented by several local studies including cohort surveys. However, at the time of the review, some gaps in the epidemiological information regarding dengue disease in Thailand were identified such as age-stratified seroprevalence data, and data relating to the proportion of hospitalized cases in the reported cases which are not easily available.

### Strengths and limitations of the review

This literature review presents the epidemiology of dengue disease in Thailand over the period 2000–2011. A key strength of this survey and analysis is that it describes the epidemiological data from a national aspect rather than from limited study site data. In addition, the review protocol aimed to minimize potential exclusions of valuable data sources including MoPH data, as well as searching for relevant books, unpublished data, abstracts and dissertations. More than 600 data sources were screened and the selected sources were subjected to a comprehensive data extraction method to capture the data, which adds strength to this review. However, by its very nature, this literature review captured mainly publicly available data and studies and is, therefore, subject to publication bias; the data presented here should be interpreted accordingly.

Another limitation of this review is that much of the peer-reviewed data are drawn from certain regions, which may skew the findings. Use of consistent MoPH data in the analysis for this review has minimized potential bias from studies using different methodologies for collecting information, confirming disease and reporting data, although this does not guarantee a consistent approach. National surveillance systems are subject to the limitations inherent to passive surveillance data, such as under-reporting, misreporting, and reporting biases. The methods and requirements for the surveillance systems in Thailand have also changed over time and the impact of the historical evolution of the systems is unknown.

In a number of papers, associations were proposed between both the burden and severity of disease and the specific DENV serotypes circulating in the population, the sequence of DENV serotypes causing primary and secondary infections or the dengue incidence in the preceding season, which indicate the multifactorial processes that influence dengue disease severity [48], [51], [52], [56], [57], [61], [63]–[66]. For example, DENV-1 has been linked with high morbidity and low mortality [61], and DSS has been associated with secondary infections attributable to DENV-2 [63]. DENV-4, which is generally found at low frequency in Southeast Asia [64], is linked to lower levels of virulence [65] and lower reported incidence [66]. Findings such as these have prompted suggestions that changes in predominant serotypes are associated with changes in disease severity [51] (see Guzman et al., 2013 for full review [41]). While such research papers have contributed to understanding the dengue disease, in the absence of nationwide data it is not clear whether the results are circumstantial (site specific) and thus it is difficult to apply the findings to other parts of the country.

### Conclusions

Dengue disease is a public health priority in Southeast Asia, and Thailand contributes substantially to the regional disease burden. Over the review period wide yearly variations in incidence occurred, with regular epidemics in 2001, 2008 and 2010 with dengue disease remaining a highly seasonal disease. Age group distribution of dengue disease shifted during the review period from younger towards older persons even if dengue disease in Thailand remain a childhood disease predominantly with higher severity reported in young children. Heterogeneous geographical patterns of the disease was observed from 2000 to 2011 including higher incidence rates reported in the South and serotype distribution variations in time and place. Passive nationwide surveillance system in Thailand is a source of consistent data including severity, age- and serotype related information. Further information on seroprevalence and on the proportion of hospitalized cases among all reported cases would be beneficial to the description and understanding of dengue epidemiology in Thailand.

## Supporting Information

Figure S1 Regions of Thailand used by the Thai Ministry of Public Health (MoPH).
(TIF)

Table S1 Databases searched for citations relating to dengue disease epidemiology in Thailand.
(PDF)

Table S2 Evidence table for citations fulfilling the inclusion and exclusion criteria for the literature review (n = 40).
(PDF)

Table S3 Incidence of dengue disease in Thailand: regional data.
(PDF)

Table S4 Age-specific patterns of dengue disease in Thailand.
(PDF)

Table S5 Serotype distribution of dengue viruses in Thailand: national and/or regional studies.
(PDF)

Checklist S1 PRISMA 2009 checklist.
(PDF)
