# Supplementary figures and images for: Epidemiological Trends of Dengue Disease in Thailand (2000–2011): A Systematic Literature Review
Source: PLoS Negl Trop Dis. 2014 Nov 6;8(11):e3241. doi: 10.1371/journal.pntd.0003241 (PMC4222696; doi:10.1371/journal.pntd.0003241)

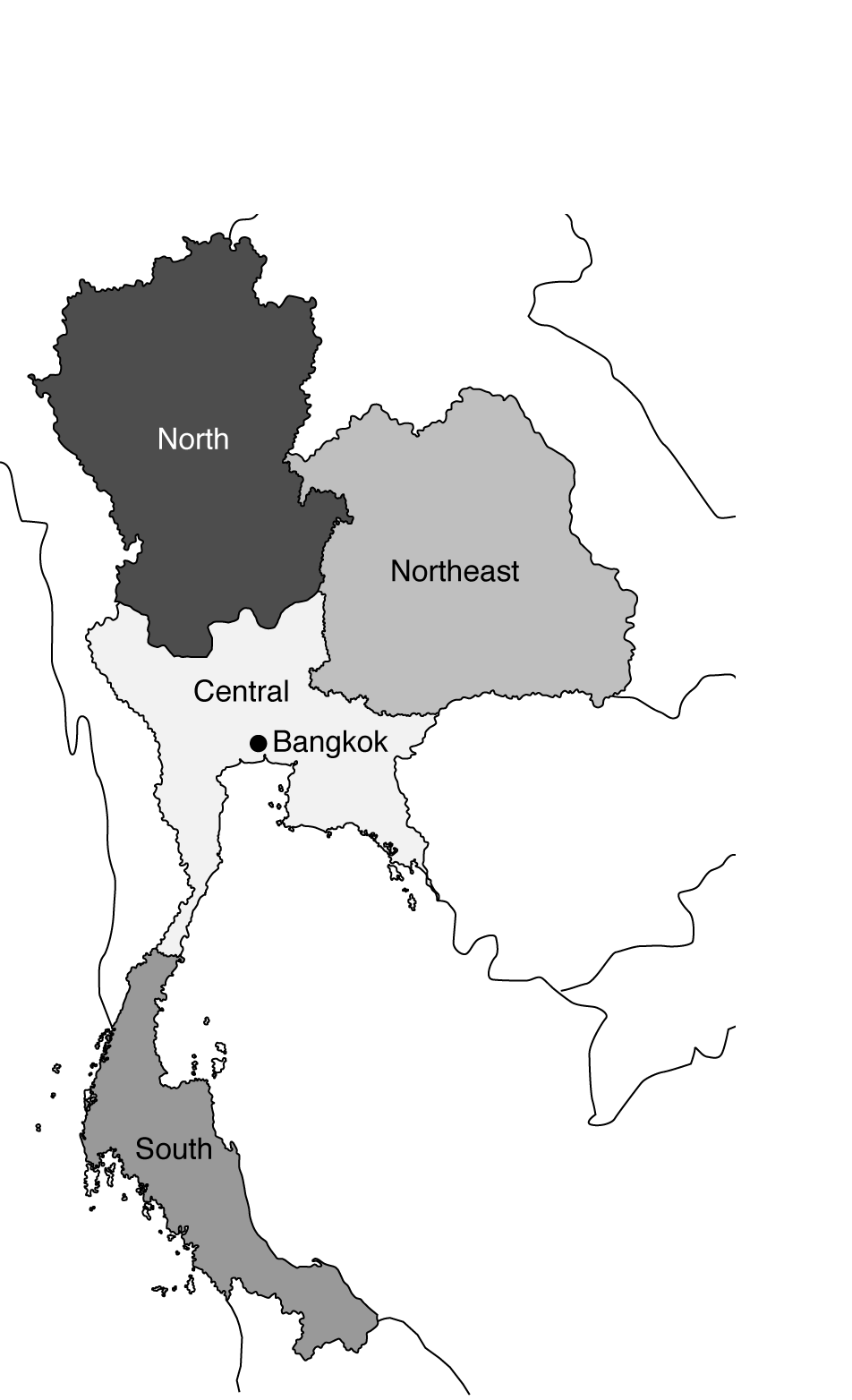

Supplement: Figure S1 — Regions of Thailand used by the Thai Ministry of Public Health (MoPH). (TIF) [file pntd.0003241.s001.tif]
