# Supplementary material for: Epidemiological Trends of Dengue Disease in Thailand (2000–2011): A Systematic Literature Review
Source: PLoS Negl Trop Dis. 2014 Nov 6;8(11):e3241. doi: 10.1371/journal.pntd.0003241 (PMC4222696; doi:10.1371/journal.pntd.0003241)
Supplement: Table S1 — Databases searched for citations relating to dengue disease epidemiology in Thailand. (PDF) [file pntd.0003241.s002.pdf]

Table S1. Databases searched for citations relating to dengue disease epidemiology in Thailand

**Database**	**Website**
Excerpta Medica Database (EMBASE)	http://embase.com
MedLine	http://www.ncbi.nlm.nih.gov/pubmed
United States National Library of Medicine and the National Institutes of Health Medical Database	http://www.ncbi.nlm.nih.gov/pubmed/
WHO Library database (WHOLIS)	http://www.who.int/publications/en/
WHO Regional Office for Southeast Asia (WHO SEAR)	http://www.searo.who.int/index.htm
Index Medicus for Southeast Asia Region (IMSEAR)	http://imsear.hellis.org/
Thailand Ministry of Health official bulletins	http://www.boe.moph.go.th
Medical Association of Thailand	http://www.mat.or.th/journal/index.php?command=vol&selvol=94
Western Pacific Surveillance and Response (WPSAR)	http://www.wpro.who.int/wpsar/home.htm
Armed Forces Research Institute of Medical Sciences	http://www.afrims.org/weblib/pub/index.shtml
WHO Western Pacific Region (WPRO)	http://www.wpro.who.int/en/
Google	
