# Supplementary material for: Epidemiological Trends of Dengue Disease in Thailand (2000–2011): A Systematic Literature Review
Source: PLoS Negl Trop Dis. 2014 Nov 6;8(11):e3241. doi: 10.1371/journal.pntd.0003241 (PMC4222696; doi:10.1371/journal.pntd.0003241)
Supplement: Table S2 — Evidence table for citations fulfilling the inclusion and exclusion criteria for the literature review (n = 40). (PDF) [file pntd.0003241.s003.pdf]

**Table S2.** Evidence table for citations fulfilling the inclusion and exclusion criteria for the literature review (n=40).

| Citation | Region/geographical area | Study design | Data period: Date range/year | If study: | | Summary of data presentation or results/conclusion |
|---|---|---|---|---|---|---|
| | | | | No. patients/population studied [M:F] | Age range | |
| MoPH, 2000–2012 [23–34] | Nationwide data | Surveillance data | 2000–2011 | | | Statistical data tables: presentation of data provided by the Thailand Ministry of Public Health |
| Kongsomboon et al., 2004 [37] | Bangkok | Observational study using an age-period cohort of temporal trends of dengue incidence rates | 1981–2000 | Not specified | 0–>15y | The study showed the age group at greatest risk for DF/DHF was 5–9 years old. The period effect shows a remittent pattern, with significant increases in 1986–1990 and 1996–2000. Authors concluded that the temporal trend of DF/DHF is decreasing; especially for DHF |
| Sriprom et al., 2003 [38] | Siriraj Hospital, Bangkok | Laboratory survey using the serological records of DHF patients. Primary and secondary infections confirmed by haemagglutination-inhibition assay (HI) test and ELISA | 1998–2003 | 1,183 patients admitted; 1,082 confirmed by serology to have DHF | <1y – >16y | Of the cases for which paired sera specimens were tested using both HI and ELISA in 2000, 2001, 2002 and the first half of 2003; six out of 48 cases (12.5%); 85 out of 293 cases (29%); 23 out of 90 cases (25.6%) and 16 out of 56 cases (28.6%), respectively, resulted from primary infections. During the last three of years of the study the proportion of primary infection above that reported between 1988 and 2003 in Bangkok (14.0%) |
| Punyagupta et al., 2009 [39] | Vichaiyut Hospital and Medical Center, Bangkok | Retrospective chart review of adult patients hospitalized at Vichaiyut Hospital and Medical Center, Bangkok, Thailand | Jan 2003 – June 2007 | 507 patients with clinical suspicion of dengue infection [248 :259] | 18y – >70y | 178 (94 males and 84 females) were confirmed as positive by either dengue IgM antibody, non-structural 1 (NS) antigen, or polymerase chain reaction (PCR). At hospitalization an initial diagnosis of dengue infection was correct in only 35 patients (46.7%). |

| Citation | Region/geographical area | Study design | Data period: Date range/year | If study: No. patients/population studied [M:F] | Age range | Summary of data presentation or results/conclusion |
|---|---|---|---|---|---|---|
| Nagao et al., 2008 [40] | North, north-eastern, central and southern regions and Bangkok | Observational study using surveillance data to examine relationship between spread of dengue is due to urbanization | 1980–2005 | Not specified | Not specified | Dengue transmission intensities in Thailand used to evaluate effect of urbanization. Inverse of mean age of dengue haemorrhagic fever (DHF) cases used as a surrogate of dengue transmission intensity. Transmission intensity in Bangkok decreased rapidly since the mid-1990s, to levels that are currently lower than in other regions. Regression analysis revealed that transmission intensity is highest in the North-eastern rural region. The results suggest that urbanization is not necessarily associated with intense dengue transmission in Thailand |
| Jianjaroonwong, 2008 [45] | Puttamonthon Hospital, Nakornpathom province | Retrospective descriptive study of patients diagnosed with dengue infection | January – December 2007 | 108 patients [57:51] | 2–46y (mean 20y) | All patients who had dengue infection were assessed for sex, age, clinical presentation, diagnosis, treatment and complication. Highest disease prevalence during July-October. Most frequent clinical features: (100%), headache (46%) , nausea/vomiting (42.6%), hematemesis (5.5%), diarrhoea (12%), and abdominal pain (9%). Severity: grade I: 38%; grade II: 14%; grade III: 3%; grade IV: 0% |
| Witayathawornwong, 2006 [46] | Petchabun province | Retrospective chart review | January 2003 – December 2005 | 627 cases of DHF were considered, from which the charts of 14 infants were reviewed [8 :6] | 3–12 months (median 8 months) | All 14 infants had signs, symptoms and laboratory investigations compatible with DHF. 5 cases (35.7%) were referred from district hospitals without definitive DHF diagnosis .11 cases (78.6%) occurred during the May−October rainy season. One 4-month-old patient had secondary dengue infection (ELISA) with DENV-4 (RT-PCR). The median duration of fever was 4.5 days; there were no deaths |

| Citation | Region/geographical area | Study design | Data period: Date range/year | If study: No. patients/population studied [M:F] | If study: Age range | Summary of data presentation or results/conclusion |
|---|---|---|---|---|---|---|
| Tipayamongkholgul et al., 2009 [47] | Southern tropical coastal provinces: Petchaburi, Prachuap Khirikhan, Chumpon, Surat Thani, Nakhon Sithammarat Northern inland mountainous provinces: Chiangmai, Lamphun, Lampang, Phrea, Nan, Phayao, Chiangrai, Maehongson | Observational study with modelling to examine the temporal relationship between El Niño and the occurrence of dengue epidemics | 1996–2005 | Total population of regions studied: Southern provinces: 3,741,656 Northern provinces: 5,746,545 Total cases of dengue between January 1996 and December 2005: Southern provinces: 54,051 Northern provinces: 44,176 | Not specified | In the two selected regions of Thailand, El Niño strength was a consistent predictor for the occurrence of dengue epidemics. Up to 22% (northern provinces) and 15% (southern provinces) of the variation in the monthly incidence of dengue cases were attributable to global ENSO cycles. In 10 of 13 provinces studied, the multivariate ENSO index was an independent predictor |
| Endy et al., 2002 [48] | Muang, a subdistrict of Kamphaeng Phet province | Prospective cohort study of children in grades 2–6 of primary schools | 1998–2000 | 1,713 children in 2000 No differences in sex distribution were noted from year to year or between schools (data not shown) | Median age: 9.3y in 2000 | 717,106 person-school days observed (1998–2000). The incidence of inapparent and symptomatic DENV infections was 1.4% and 0.8% in 2000, respectively; 1.1% of acute-illness school absences in 2000 were attributed to symptomatic DENV infection. The results illustrate the spatial and temporal diversity of dengue virus infection and the burden of dengue disease in schoolchildren in Thailand |
| Khawsak et al., 2003 [49] | Central (Bangkok; Samut Prakan; Nakhon Sawan; Pathum Thani, Ratchaburi, Nakhon Pathom, Samut Songkhram); Northeast (Ubon Ratchathani, Udon Thani); North (Chiang Rai, Phitsanulok); South (Ranong; Nakhon Si Thammarat, Yala) | Retrospective laboratory serotype analysis usinga modified reverse transcriptase-polymerase chain reaction (RT-PCR) and a single-tube multiplex PCR assay | 2000–2001 | 637 blood samples. Male/female breakdown not given | Not specified | 71, 43, 28, and 43 patients were classified as having a single infection with serotypes 1, 2, 3, and 4, respectively.DENV-1 was abundant in Bangkok metropolitan area; serotypes 2, 3, and 4 were dominant in suburban areas. The percentage of single infection was approximately 29% (185 out of 637) |

| Citation | Region/geographical area | Study design | Data period: Date range/year | If study: No. patients/population studied [M:F] | If study: Age range | Summary of data presentation or results/conclusion |
|---|---|---|---|---|---|---|
| Anantapreecha et al., 2004 [50] | Hospitals in Lampang, Nakhon Ratchasima, Pathum Thani, Bangkok, Ratchaburi and Songkhla provinces. | Serotype | 1999–2002 | Blood samples collected from suspected dengue cases at acute (n= 5,160) and convalescent (n=3,619) stages | Not specified | All four dengue serotypes were identified; overall DENV-1 was the predominant serotype (48%), followed by DENV-2 (27%), DENV-3 (20%) and DENV-4 (5%).The predominant dengue serotypes changed every 1-2 years in three of the six hospitals and the predominant serotypes were different in different hospitals |
| Veeraseatakul et al., 2007 [51] | Five northern provinces (Chiangmai, Lampang, Lamphun, Mae Hong Son, Phrae) | Clinical study to determine the dengue serotypes in circulation using RT-PCR | 2002–2006 | 1,116 seropositive acute samples were analysed from DF/DHF patients | Not specified | 559 samples were DENV positive: DENV-2: 47.2%; DENV-1: 30.6%; DENV-4: 18.4%; DENV-3: 3.8%. DENV-2 was the predominant dengue serotype from 2002–2005; DENV-1 was predominant in 2006. There was an apparent increase in the percentage of DENV-4 from 2005 to 2006 |
| Fried et al., 2010 [52] | Queen Sirikit Institute of Child Health, Bangkok | Prospective observational single-site study to determine associations between serotype and signs of severe clinical disease | 1994–2006 | 457 patients [254:204] | 18mo –15y; mean age 8.6y | Data for the whole period reveal 162 (36%) cases with DENV-1, 102 (23%) with DENV-2, 123 (27%) with DENV-3, and 64 (14%) with DENV-4. Comparing study years, the rate of DHF was significantly less in 1999, 2000, 2004, and 2005 than in 1994, the study year with the highest percentage of DHF cases. There was no significant difference in the rates of DHF by serotype |

| Citation | Region/geographical area | Study design | Data period: Date range/year | If study: | | Summary of data presentation or results/conclusion |
|---|---|---|---|---|---|---|
| | | | | No. patients/population studied [M:F] | Age range | |
| Limkittikul et al., 2005 [53] | Northeast region provincial hospitals | Retrospective study to compare the clinical spectra of disease in patients with confirmed dengue. | June–September 2002 | Medical records of 99 dengue-infected in-patients, confirmed by PCR and paired antidengue IgG and IgM ELISA tests, were examined. | 3–30 years. | Infecting serotypes: DENV-1 (21%), DENV-2 (55%), DENV-3 (12%) or DENV-4 (12%): 22% primary and 78% secondary infections. Dengue fever was the most common presentation for both primary (77.2%) and secondary infections (46.7%). The DF:DHF and non-DSS:DSS ratios were lowest for DENV-2. There was no difference in the duration of fever, percentage of hepatomegaly and bleeding among the serotypes |
| Anantapreecha et al., 2007 [54] | Hospitals in Lampang, Nakhon Ratchasima, Pathum Thani, Bangkok, Ratchaburi and Songkhla provinces | Antibody response study in patients with confirmed primary dengue virus infection who visited the selected hospitals | 1999–2002 | Acute and convalescent plasma samples obtained from 101 confirmed primary dengue cases | Not specified | DENV-1: 48 cases; DENV-2: 10 cases; DENV-3: 42 cases; DENV-4: 1 case. Haemagglutination inhibition titres of individual samples were similar at both acute and convalescent stages, irrespective of DENV serotype. The results indicate that haemagglutination inhibition antibodies to DENV are cross-reactive, which should be considered when it is used as a diagnostic test |
| Anantapreecha et al., 2005 [55] | Hospitals in Lampang, Nakhon Ratchasima, Pathum Thani, Bangkok, Ratchaburi and Songkhla provinces | Serological and virological analysis: DF and DHF | 1999–2002 | 2,715 serologically and virologically confirmed clinical diagnoses of DF or DHF | <1y to ≥66y | DENV-1: 45%; DENV-2: 32%; DENV-3: 18%; DENV-4: 5%. Majority of DHF cases caused by DENV-2 and DENV-4 were secondary infections; approximately 20% of DHF cases caused by DENV-1 and DENV-3 were primary infections. Male:female ratios and age distribution were similar among the four serotypes in either primary or secondary infections |

| Citation | Region/geographical area | Study design | Data period: Date range/year | If study: No. patients/population studied [M:F] | If study: Age range | Summary of data presentation or results/conclusion |
|---|---|---|---|---|---|---|
| Endy et al., 2011 [56] | Kamphaeng Phet, northern Thailand | Prospective study in schoolchildren describing spatial and temporal variations in the symptomatic to inapparent (S:I) illness ratio | 1998–2002 | There was a gradual decline of the study population over time: 2,044 in 1998; 1,915 in 1999; 2,203 in 2000; 2,011 in 2001; and 1,759 in 2002 | 7–16y | 1,024 dengue virus infections were detected in total over the 5 years of the study; 909 children experienced at least one dengue infection during the study period and 115 experienced a second infection. There was a wide fluctuation in the S:I illness ratio between and among schools in a given year and within schools over several dengue seasons. For a given school, the most important determinants of S:I ratio were the incidence of dengue infection in a given year and in the preceding year |
| Poblap et al., 2006 [58] | Central Plain region Nakhon Pathom province; 15 villages in the Museang and Sam Phran districts | Cross-sectional clinical study using active serological surveys and viral RNA isolation to detect silent transmission of dengue virus | 27 March – 11 April 2001 | 329 healthy volunteers | 0 to >50y | Twenty-nine sera tested positive for dengue IgM (8.8%) and one for Japanese encephalitis IgM. Of those positive for dengue IgM antibody 13.7% (23/168) were males and 7.5% (12/161) were females, with no significant difference between the two. The highest prevalence occurred in the 15–40-year-old group. Viral RNA was detected by PCR in 2 cases (DENV-3 was identified) |

| Citation | Region/geographical area | Study design | Data period: Date range/year | If study: | | Summary of data presentation or results/conclusion |
|---|---|---|---|---|---|---|
| | | | | No. patients/population studied [M:F] | Age range | |
| Mammen et al., 2008 [59] | Muang district, Kamphaeng Phet province, north-central Thailand | Prospective cluster study, during two seasons of peak DENV transmission to test the hypothesis that DENV transmission is spatially and temporally focal | 2004–2005 | 1,204 febrile children (506 in 2004 and 698 in 2005)<br>48 had detectable DENV viremia (28 in 2004 and 20 in 2005) | 6 momths–15y | Study compared geographic and temporal characteristics within Thai villages where DENV were not being actively transmitted. Thirty-four cluster investigations were conducted during the study period. The data reveal the remarkably focal nature of DENV transmission within a hyperendemic rural area of Thailand. Ten clusters (five pairs) in 2004 and two clusters (one pair) in 2005 were spatially and temporally matched. Children in 58% (seven of 12) of the positive clusters (six in 2004 and one in 2005) attended a single school |
| Tuntaprasart et al., 2003 [60] | Muenag district, Ratchaburi province | Seroepidemiological study | July 2000 – June 2001 | 341 schoolchildren included in survey: 283 specimens tested | 5–12y | Of the tested specimens, 71% (200/283) were positive for dengue IgG antibody at baseline (July 2000). 90 schoolchildren were followed up three times, in September and December 2000, and June 2001. In June 2001, the rate of dengue infection showed an increase of 8.8% with 8.0% among immune children and 10.3% among naïve schoolchildren. The rate of seroconversion increased between September and December 2000 |
| Choudhri et al., 2011 [61] | Nationwide | WHO Report of national surveillance data from countries in the WHO South-East Asia Region | 2000–2010 | | | Situation update of dengue in the South-East Asia Region, 2010. Reported number of cases of DF/ DHF in the South-East Asia Region, including Thailand, 2000–2010 |
| Wichmann et al., 2011 [62] | Muang district of Kamphaeng Phet province and Muang district of Ratchaburi province | Observational cohort studyby members of a dengue field site consortium over at least two dengue seasons | 2003–2006 | 14,627 person-years of prospective cohort data were obtained | <15y | Cohort studies were conducted among children aged <15 years by members of a dengue field site consortium over at least 2 dengue seasons. Average under-recognition of total and inpatient dengue cases was 8.7 and 2.6-fold in Thailand, and 9.1 and 1.4-fold in Cambodia, respectively |

| Citation | Region/geographical area | Study design | Data period: Date range/year | If study: No. patients/population studied [M:F] | If study: Age range | Summary of data presentation or results/conclusion |
|---|---|---|---|---|---|---|
| Klungthong et al., 2004 [64] | Bangkok | Molecular epidemiology study of genetic diversity and evolutionary change of DENV-4 | 1973–2002 | 53 dengue viral isolates recovered | Not specified | Study revealed extensive genetic diversity within a single locality at a single time. A new and divergent genotype of DENV-4 was discovered and a pattern of continual lineage turnover observed. There was no evidence for adaptive evolution in any gene, codon, or lineage of DENV-4 |
| Silawan et al., 2008 [Suppl. Table S3: Ref. A] | Nakhon Ratchasima, Buri Ram, Surin, Si Sa Ket, Ubon Ratchathani, Yasothon, Chaiyaphum, Amnat Charoen, Nong Bua Lam Phu, Khon Kaen, Udon Thani, Loei, Nong Khai, Maha Sarakham, Roi Et, Kalasin, Sakon Nakhon, Nakhon Phanom, Mukdahan | Observational modelling study | 1996–2005; predictions 2006–2008 | Regional census population, 21,297,769 | Not specified | The forecasting model for dengue incidence was evaluated by comparing predicted versus actual rates of dengue for 1996−2005 and used to forecast monthly rates during January−December 2006. Epidemics occurred every 2 years |
| Ellis et al., 2006 [Suppl. Table S3: Ref. B] | Sangkhlaburi district, Kanchanaburi province, western Thailand | Prospective clinical study | June 1999 – March 2002 | 613 adult inpatients and outpatients evaluated for cause of fever .[325:288] | 20–87y (median age 38y) | An etiologic diagnosis was made based on clinical findings and laboratory results in 48% of cases. Malaria was the most common diagnosis (25%). Serologic evidence for leptospirosis was found in 17% of cases. Other aetiologic diagnoses included rickettsial infections, dengue fever, and typhoid |
| Anderson et al., 2007 [Suppl. Table S3: Ref. C] | Kamphaeng Phet province, northern Thailand | Prospective cohort study of primary schoolchildren to assess disability-adjusted life years (DALYs) and patient costs per illness | 1998–2002 | 8807 children in total (1998–2002). Of these, n = 1,713, 1,575 1,372 and in 2000, 2001 and 2002, respectively | 5–15y | Dengue accounted for 328 (11%) of the 3056 febrile cases identified in 2114 children during the study period. The mean burden of dengue was 465·3 DALYs/million population/year. Non-hospitalized patients with dengue illnesses represented a substantial proportion of the overall burden of disease. DALYs lost to dengue each year and dengue incidences for each year of the study were presented |

| Citation | Region/geographical area | Study design | Data period: Date range/year | If study: | | Summary of data presentation or results/conclusion |
|---|---|---|---|---|---|---|
| | | | | No. patients/population studied [M:F] | Age range | |
| Promprou et al., 2006 [Suppl. Table S3: Ref. D] | Southern Thailand | Observational study using univariate time-series analysis to model and forecast the monthly number of DHF cases | 1994–2005 | Not specified | Not specified | Autoregressive integrated moving average (ARIMA) models were developed using data collected between 1994 and 2005 and validated using data collected between January and August 2006. The results showed that the time-series forecasting of DHF cases in southern Thailand may help improve public health planning for disease prevention and control |
| Sriprom et al., 2010 [Suppl. Table S3: Ref. E] | Sakon Nakhon Province (18 districts) | Observational study using a statistical and autoregressive analysis to construct district level maps of the distribution of dengue infection | January 2005 – December 2007 | 1,894 hospitalised patients diagnosed with dengue infection 985 males; 909 females | 4 months – 65y | High correlation between dengue incidence and weather conditions (monthly minimum temperature, past 2-month cumulated rainfall) and socio-economical (population of 0–4 years old, per capita number of public small water wells, proportion of villages with primary schools) covariates. Higher occurrences observed in the three most populated districts (Wanon Niwat, Sawang Daen Din and Mueang Sakon Nakhon) |
| Ditsuwan et al., 2011 [Suppl. Table S3: Ref. F] | Provinces in lower southern Thailand (Narathiwat, Pattani, Yala, Songkhla, Trang, Phatthalung, Satun) | Descriptive surveillance study to assess the incidence of dengue infection | August 2008 – June 2009 | 27,166 patients identified, including 3,319 with dengue infection [1,776; 1,543 (Calculated using percentages given in text and table)] | Mean: 19.2y ± 15.3 | Dengue incidence: 73 /100,000 population. Of those with dengue, 1447 (43.6%) and 1827 (55.0%) were DF and DHF, respectively. 45 dengue cases were diagnosed as DSS; of these, 3 died. Most patients (85.5%) lived in rural areas; 75.4% of cases were admitted to hospital |
