# Supplementary material for: Epidemiological Trends of Dengue Disease in Thailand (2000–2011): A Systematic Literature Review
Source: PLoS Negl Trop Dis. 2014 Nov 6;8(11):e3241. doi: 10.1371/journal.pntd.0003241 (PMC4222696; doi:10.1371/journal.pntd.0003241)
Supplement: Table S3 — Incidence of dengue disease in Thailand: regional data. (PDF) [file pntd.0003241.s004.pdf]

Table S3. Incidence of dengue disease in Thailand: regional data.

Year	Region	DF		Laboratory-confirmed DF (n)	DHF (n)	DF-related mortality (n)	Source of data
		Notified cases (n)	Incidence (per 100,000 population)				
1996–2005	Amnat Charoen		3.19*				Silawan et al., 2008[A]
1996–2005	Buri Ram		4.58*				Silawan et al., 2008[A]
1996–2005	Chaiyaphum		3.63*				Silawan et al., 2008[A]
1996–2005	Kalasin		3.75*				Silawan et al., 2008[A]
1996–2005	Khon Kaen		3.19*				Silawan et al., 2008[A]
1996–2005	Loei		3.35*				Silawan et al., 2008[A]
1996–2005	Maha Sarakham		1.94*				Silawan et al., 2008[A]
1996–2005	Mukdahan		3.92*				Silawan et al., 2008[A]
1996–2005	Nakhon Phanom		1.77*				Silawan et al., 2008[A]
1996–2005	Nakhon Ratchasima		4.63*				Silawan et al., 2008[A]
1996–2005	Nong Bua Lam Phu		2.13*				Silawan et al., 2008[A]
1996–2005	Nong Khai		2.76*				Silawan et al., 2008[A]
1996–2005	Roi Et		4.17*				Silawan et al., 2008[A]
1996–2005	Sakon Nakhon		4.01*				Silawan et al., 2008[A]
1996–2005	Si Sa Ket		4.00*				Silawan et al., 2008[A]
1996–2005	Surin		3.61*				Silawan et al., 2008[A]
1996–2005	Ubon Ratchathani		3.93*				Silawan et al., 2008[A]
1996–2005	Udon Thani		2.07*				Silawan et al., 2008[A]
1996–2005	Yasothon		2.30*				Silawan et al., 2008[A]
1996–2005	Northeast region (total)		3.53*				Silawan et al., 2008[A]
1996–2005	Northern region	44,176					Tipayamongkholgul et al., 2009 [47]
1996–2005	Southern region	54,051					Tipayamongkholgul et al., 2009 [47]

Year	Region	DF		Laboratory-confirmed DF (n)	DHF (n)	DF-related mortality (n)	Source of data
		Notified cases (n)	Incidence (per 100,000 population)				
1996–2005	Chiangmai		~6*†				Tipayamongkholgul et al., 2009 [47]
1996–2005	Chiangrai		~6*†				Tipayamongkholgul et al., 2009 [47]
1996–2005	Chumphon		~10*†				Tipayamongkholgul et al., 2009 [47]
1996–2005	Lampang		~8*†				Tipayamongkholgul et al., 2009 [47]
1996–2005	Lampun		~6*†				Tipayamongkholgul et al., 2009 [47]
1996–2005	Maehongson		~4*†				Tipayamongkholgul et al., 2009 [47]
1996–2005	Nakhon Sithammarat		~10*†				Tipayamongkholgul et al., 2009 [47]
1996–2005	Nan		~4*†				Tipayamongkholgul et al., 2009 [47]
1996–2005	Petchaburi		~9*†				Tipayamongkholgul et al., 2009 [47]
1996–2005	Phayao		~8*†				Tipayamongkholgul et al., 2009 [47]
1996–2005	Phrae		~7*†				Tipayamongkholgul et al., 2009 [47]
1996–2005	Prachuap Khirikhan		~11*†				Tipayamongkholgul et al., 2009 [47]
1996–2005	Surat Thani		~18*†				Tipayamongkholgul et al., 2009 [47]

Year	Region	DF		Laboratory-confirmed DF (n)	DHF (n)	DF-related mortality (n)	Source of data
		Notified cases (n)	Incidence (per 100,000 population)				
1999–2002	Thai–Myanmar border[‡]			9			Ellis et al., 2006[B]
2000	Bangkok[§]				71		Sriprom et al., 2003 [38]
2000	Bangkok[§]			24	7[¶]		Fried et al., 2010 [52]
2000	Kamphaeng Phet					0	Endy et al., 2011 [56]
2000	Kamphaeng Phet**			15			Anderson et al., 2007[C]
2000	Central region				13,013	18	MoPH, 2002 [25]
2000	Northern region				1751	4	MoPH, 2002 [25]
2000	Northeastern region				1648	1	MoPH, 2002 [25]
2000	Southern region				2205	9	MoPH, 2002 [25]
2000–2004	Southern Thailand[††]				113,591	251	Promprou et al., 2006[D]
2001	Bangkok[‡‡]				334		Sriprom et al., 2003 [38]
2001	Bangkok[‡‡]			27	18**		Fried et al., 2010 [52]
2001	Kamphaeng Phet					0	Endy et al., 2011 [56]
2001	Kamphaeng Phet**			132		0	Anderson et al., 2007[C]
2001	Central region				57,567	106	MoPH, 2002 [25]
2001	Northern region				27,562	33	MoPH, 2002 [25]
2001	Northeastern region				27,668	42	MoPH, 2002 [25]
2001	Southern region				26,558	64	MoPH, 2002 [25]
2002	Bangkok[§]				186		Sriprom et al., 2003 [38]
2002	Bangkok[§]			16	9**		Fried et al., 2010 [52]
2002	Kamphaeng Phet						Endy et al., 2011 [56]
2002	Kamphaeng Phet**			33			Anderson et al., 2007[C]
2002	Central region				30,027	32	MoPH 2002 [25]
2002	Northern region				13,915	23	MoPH 2002 [25]
2002	Northeastern region				37,191	57	MoPH 2002 [25]

Year	Region	DF		Laboratory-confirmed DF (n)	DHF (n)	DF-related mortality (n)	Source of data
		Notified cases (n)	Incidence (per 100,000 population)				
2002	Southern region				33,667	64	MoPH 2002 [25]
2002	Southern Thailand[††]				33,617	64; 0.77	Promprou et al., 2006[D]
2003	Bangkok[§‡‡]				121	0	Sriprom et al., 2003 [38]
2003	Central region			25,406			MoPH, 2003 [26]
2003	Northern region			11,092			MoPH, 2003 [26]
2003	Northeastern region			18,954			MoPH, 2003 [26]
2003	Southern region			8205			MoPH, 2003 [26]
2004	Bangkok[§]			36	14**		Fried et al., 2010 [52]
2005	Southern Thailand[†‡‡]				2991	5	Promprou et al., 2006[D]
2005	Akat Amnuai	5					Sriprom et al., 2010[E]
2005	Ban Muang	18					Sriprom et al., 2010[E]
2005	Charoen Sin	10					Sriprom et al., 2010[E]
2005	Kham Ta Kla	7					Sriprom et al., 2010[E]
2005	Khok Si Suphan	20					Sriprom et al., 2010[E]
2005	Ku Su Man	8					Sriprom et al., 2010[E]
2005	Kut Bak	6					Sriprom et al., 2010[E]
2005	Mueang Sakon Nakhon	75					Sriprom et al., 2010[E]
2005	Nikhom Nam Un	4					Sriprom et al., 2010[E]
2005	Phang Khon	12					Sriprom et al., 2010[E]
2005	Phanna Nikhom	11					Sriprom et al., 2010[E]
2005	Phon Na Kaeo	4					Sriprom et al., 2010[E]
2005	Phu Phan	14					Sriprom et al., 2010[E]
2005	Sawang Daen Din	54					Sriprom et al., 2010[E]
2005	Song Dao	9					Sriprom et al., 2010[E]
2005	Tao Ngoi	17					Sriprom et al., 2010[E]
2005	Wanon Niwat	77					Sriprom et al., 2010[E]

Year	Region	DF		Laboratory-confirmed DF (n)	DHF (n)	DF-related mortality (n)	Source of data
		Notified cases (n)	Incidence (per 100,000 population)				
2005	Waritchaphum	11					Sriprom et al., 2010[E]
2005	Sakon Nakhon province	362					Sriprom et al., 2010[E]
2005	Bangkok[§]			25	7[¶]		Endy et al., 2011 [56]
2006	Akat Amnuai	15					Sriprom et al., 2010[E]
2006	Ban Muang	44					Sriprom et al., 2010[E]
2006	Charoen Sin	13					Sriprom et al., 2010[E]
2006	Kham Ta Kla	40					Sriprom et al., 2010[E]
2006	Khok Si Suphan	7					Sriprom et al., 2010[E]
2006	Ku Su Man	15					Sriprom et al., 2010[E]
2006	Kut Bak	48					Sriprom et al., 2010[E]
2006	Mueang Sakon Nakhon	114					Sriprom et al., 2010[E]
2006	Nikhom Nam Un	8					Sriprom et al., 2010[E]
2006	Phang Khon	12					Sriprom et al., 2010[E]
2006	Phanna Nikhom	11					Sriprom et al., 2010[E]
2006	Phon Na Kaeo	16					Sriprom et al., 2010[E]
2006	Phu Phan	12					Sriprom et al., 2010[E]
2006	Sawang Daen Din	142					Sriprom et al., 2010[E]
2006	Song Dao	105					Sriprom et al., 2010[E]
2006	Tao Ngoi	6					Sriprom et al., 2010[E]
2006	Wanon Niwat	20	4				Sriprom et al., 2010[E]
2006	Waritchaphum	10					Sriprom et al., 2010[E]
2006	Sakon Nakhon province	822					Sriprom et al., 2010[E]
2006	Bangkok[§]			24	20[¶]		Endy et al., 2011 [56]
2007	Akat Amnuai	64					Sriprom et al., 2010[E]
2007	Ban Muang	25					Sriprom et al., 2010[E]
2007	Charoen Sin	1					Sriprom et al., 2010[E]

Year	Region	DF		Laboratory-confirmed DF (n)	DHF (n)	DF-related mortality (n)	Source of data
		Notified cases (n)	Incidence (per 100,000 population)				
2007	Kham Ta Kla	90					Sriprom et al., 2010[E]
2007	Khok Si Suphan	14					Sriprom et al., 2010[E]
2007	Ku Su Man	7					Sriprom et al., 2010[E]
2007	Kut Bak	0					Sriprom et al., 2010[E]
2007	Mueang Sakon Nakhon	81					Sriprom et al., 2010[E]
2007	Phang Khon	11					Sriprom et al., 2010[E]
2007	Phanna Nikhom	9					Sriprom et al., 2010[E]
2007	Phon Na Kaeo	11					Sriprom et al., 2010[E]
2007	Phu Phan	21					Sriprom et al., 2010[E]
2007	Sawang Daen Din	54					Sriprom et al., 2010[E]
2007	Song Dao	22					Sriprom et al., 2010[E]
2007	Tao Ngoi	6					Sriprom et al., 2010[E]
2007	Wanon Niwat	12					Sriprom et al., 2010[E]
2007	Waritchaphum	17					Sriprom et al., 2010[E]
2007	Sakon Nakhon province	265					Sriprom et al., 2010[E]
2007	Nakornpathom[§]				108	0	Jianjaroonwong 2008 [45]
2007	Central region		129.34				MoPH, 2007 [30]
2007	Northern region		73.25				MoPH, 2007 [30]
2007	Northeastern region		83.05				MoPH, 2007 [30]
2007	Southern region		137.96				MoPH, 2007 [30]
2008	Ang Thong		280.73				MoPH, 2008 [31]
2008	Chieng Mai		255.55				MoPH, 2008 [31]
2008	Nakorn Pathom		303.84				MoPH, 2008 [31]
2008	Nakorn Sawan		245.63				MoPH, 2008 [31]
2008	Phuket		244.05				MoPH, 2008 [31]
2008	Rachaburi		383.25				MoPH, 2008 [31]

Year	Region	DF		Laboratory-confirmed DF (n)	DHF (n)	DF-related mortality (n)	Source of data
		Notified cases (n)	Incidence (per 100,000 population)				
2008	Rayong		355.29				MoPH, 2008 [31]
2008	Samut Prakarn		278.17				MoPH, 2008 [31]
2008	Samut Sakorn		321.07				MoPH, 2008 [31]
2008	Utaradit		271.55				MoPH, 2008 [31]
2008–2009[§§]	Lower Southern Thailand		1447		1827		Ditsuwan et al., 2011[F]
2009	Krabi		206.8				MoPH, 2009 [32]
2009	Mae Hong Son		244.27				MoPH, 2009 [32]
2009	Pang Nga		268.35				MoPH, 2009 [32]
2009	Pataloong		143.55				MoPH, 2009 [32]
2009	Prae		146.29				MoPH, 2009 [32]
2009	Rachaburi		189.46				MoPH, 2009 [32]
2009	Rayong		157.59				MoPH, 2009 [32]
2009	Samut Prakarn		176.69				MoPH, 2009 [32]
2009	Songkla		150.99				MoPH, 2009 [32]
2009	Tak		179.96				MoPH, 2009 [32]
2010	Chanthaburi		433.2				MoPH, 2010 [33]
2010	Chiang Mai		400				MoPH, 2010 [33]
2010	Krabi		390.57				MoPH, 2010 [33]
2010	Nakhon Si		397.43				MoPH, 2010 [33]
2010	Narathiwat		475.69				MoPH, 2010 [33]
2010	Pattani		490.6				MoPH, 2010 [33]
2010	Phatthalung		408.53				MoPH, 2010 [33]
2010	Satun		359.84				MoPH, 2010 [33]
2010	Songkhla		447.84				MoPH, 2010 [33]
2010	Tak		399.29				MoPH, 2010 [33]

Empty cells denote no data are available. DF, dengue fever; DHF, dengue haemorrhagic fever; MoPH, Thai Ministry of Public Health.

*Monthly incidence rate.
[†]Estimated from graphs.
[‡]June 1999 to March 2002.
[§]Data from one hospital (Kamphaeng Phet Field Station).
[¶]Patients with serological data available.
**Children aged 5–15 years only.
[††]Data from Introduction of paper.
[‡‡]Data for January to June.
[§§]August 2008 to June 2009.

References

A. Silawan T, Singhasivanon P, Kaewkungwal J, Nimmanitya S, Suwonkerd W (2008) Temporal patterns and forecast of dengue infection in Northeastern Thailand. Southeast Asian J Trop Med Public Health 39(1): 90–98.

B. Ellis RD, Fukuda MM, McDaniel P, Welch K, Nisalak A, et al. (2006) Causes of fever in adults on the Thai–Myanmar border. Am J Trop Med Hyg 74(1): 108–113.

C. Anderson KB, Chunsuttiwat S, Nisalak A, Mammen MP, Libraty DH, et al. (2007) Burden of symptomatic dengue infection in children at primary school in Thailand: a prospective study. Lancet 369(9571): 1452–1459.

D. Promprou S, Jaroensutasinee M, Jaroensutasinee K (2006) Forecasting dengue haemorrhagic fever cases in southern Thailand using ARIMA models. Dengue Bull 30: 99–106.

E. Sriprom M, Chalvet-Monfray K, Chaimane T, Vongsawat K, Bicout DJ (2010) Monthly district level risk of dengue occurrences in Sakon Nakhon Province, Thailand. Sci Total Environ; 408(22): 5521–5528.

F. Ditsuwan T, Liabsuetrakul T, Chongsuvivatwong V, Thammapalo S, McNeil E (2011) Assessing the spreading patterns of dengue infection and chikungunya fever outbreaks in lower southern Thailand using a geographic information system. Ann Epidemiol 21(4): 253–261.
