# Supplementary material for: Epidemiological Trends of Dengue Disease in Thailand (2000–2011): A Systematic Literature Review
Source: PLoS Negl Trop Dis. 2014 Nov 6;8(11):e3241. doi: 10.1371/journal.pntd.0003241 (PMC4222696; doi:10.1371/journal.pntd.0003241)
Supplement: Table S4 — Age-specific patterns of dengue disease in Thailand. (PDF) [file pntd.0003241.s005.pdf]

Table S4. Age-specific patterns of dengue disease in Thailand [18, 23–34].

Characteristic	Age group (years)										Total
	0–4	5–9	10–14	15–24	25–34	35–44	45–54	55–64	65+	Unknown age	
**2000**											
DSS (27), n											
DHF (26), n											
DF (66), n											
Total dengue disease (26,27,66), n	2758	6181	5260	2744	964	472	137	57	44	0	18,617
Deaths, n	8	11	9	2	2	0	0	0	0	0	32
Population, n	5,173,354	5,340,120	5,411,127	11,340,156	10,746,046	9,141,069	6,562,362	4,397,633	3,658,392		61,770,259
Incidence, /100,000	53.31	115.75	97.21	24.20	8.97	5.16	2.09	1.30	1.20		30.14
CFR	0.29	0.18	0.17	0.07	0.21	0.00	0.00	0.00	0.00		0.17
**2001**											
DSS (27), n											
DHF (26), n											
DF (66), n											
Total dengue disease (26,27,66), n	16,952	43,813	40,213	24,897	7835	3500	1380	491	274	0	139,355
Deaths, n	34	91	53	39	16	5	4	2	1	0	245
Population, n	5,125,593	5,293,426	5,371,420	11,247,513	10,810,122	9,251,874	6,806,804	4,429,535	3,757,568		62,093,855
Incidence, /100,000	330.73	827.69	748.65	221.36	72.48	37.83	20.27	11.08	7.29		224.43
CFR	0.20	0.21	0.13	0.16	0.20	0.14	0.29	0.41	0.36		0.18
**2002**											
DSS (27), n	268	1195	1053	260	61	42	15	12	12	0	2918
DHF (26), n	7092	21,666	24,097	16,820	5322	2416	1028	419	255	38	79,153
DF (66), n	4032	10,473	10,104	5011	1682	837	360	128	99	3	32,729

Characteristic	Age group (years)										Total
	0–4	5–9	10–14	15–24	25–34	35–44	45–54	55–64	65+	Unknown age	
Total dengue disease (26,27,66), n	11,392	33,334	35,254	22,091	7065	3295	1403	559	366	41	114,800
Deaths, n	27	68	37	29	6	7	2	0	0	0	176
Population, n	4,291,010	5,012,944	4,846,628	10,578,583	11,603,961	10,462,603	7,099,944	4,278,967	4,379,761		62,554,401
Incidence, /100,000	265.49	664.96	727.39	208.83	60.88	31.49	19.76	13.06	8.36		183.52
CFR	0.24	0.20	0.10	0.13	0.08	0.21	0.14	0.00	0.00		0.15
**2003**											
DSS (27), n	108	493	447	128	42	14	13	17	24	2	1288
DHF (26), n	3229	10,765	13,443	10,493	3800	1838	793	306	150	60	44,877
DF (66), n	1865	5198	5561	2977	968	511	233	109	65	5	17,492
Total dengue disease (26,27,66), n	5202	16,456	19,451	13,598	4810	2363	1039	432	239	67	63,657
Deaths, n	10	21	14	3	4	0	0	0	0	0	52
Population, n	4,133,411	4,982,438	4,922,276	10,371,683	11,501,542	10,700,519	7,415,280	4,384,319	4,528,351		62,939,819
Incidence, /100,000	125.85	330.28	395.16	131.11	41.82	22.08	14.01	9.85	5.28		101.14
CFR	0.19	0.13	0.07	0.02	0.08	0.00	0.00	0.00	0.00		0.08
**2004**											
DSS (27), n	68	294	322	101	24	15	6	9	3	1	843
DHF (26), n	1737	5808	7916	6836	2605	1316	544	171	140	38	27,111
DF (66), n	1028	3134	3650	2032	719	348	173	64	30	3	11,181
Total dengue disease (26,27,66), n	2833	9236	11,888	8969	3348	1679	723	244	173	42	39,135
Deaths, n	7	10	6	6	2	3	0	1	0	0	35
Population, n	4,133,411	4,982,438	4,922,276	10,371,683	11,501,542	10,762,684	7,640,375	4,433,108	4,573,101		63,320,618
Incidence, /100,000	68.54	185.37	241.51	86.48	29.11	15.60	9.46	5.50	3.78		61.80
CFR	0.25	0.11	0.05	0.07	0.06	0.18	0.00	0.41	0.00		0.09

Characteristic	Age group (years)										Total
	0–4	5–9	10–14	15–24	25–34	35–44	45–54	55–64	65+	Unknown age	
**2005**											
DSS (27), n	75	293	323	115	51	23	15	11	16	0	922
DHF (26), n	1804	5656	8157	8184	3312	1588	708	255	179	58	29,901
DF (66), n	1241	3890	4637	3090	1154	607	279	104	60	8	15,070
Total dengue disease (26,27,66), n	3120	9839	13,117	11,389	4517	2218	1002	370	255	66	45,893
Deaths, n	5	15	8	7	9	3	1	1	1	0	50
Population, n	4,033,986	4,833,182	4,905,179	10,094,340	11,250,738	10,753,119	7,861,649	4,553,211	4,608,850		62,894,254
Incidence, /100,000	77.34	203.57	267.41	112.83	40.15	20.63	12.75	8.13	5.53		72.97
CFR	0.16	0.15	0.06	0.06	0.20	0.14	0.10	0.27	0.39		0.11
**2006**											
DSS (27), n	39	247	287	113	31	15	2	4	5	0	743
DHF (26), n	1482	5267	8291	8039	3103	1685	670	265	171	51	29,024
DF (66), n	1262	4345	5731	3476	1125	601	314	122	73	13	17,062
Total dengue disease (26,27,66), n	2783	9859	14,309	11,628	4259	2301	986	391	249	64	46,829
Deaths, n	3	15	8	11	5	2	0	0	0	0	44
Population, n	3,991,348	4,649,267	4,871,031	9,855,993	11,051,410	10,878,282	8,154,852	4,755,480	4,737,730		62,945,393
Incidence, /100,000	69.73	212.05	293.76	117.98	38.54	21.15	12.09	8.22	5.26		74.40
CFR	0.11	0.15	0.06	0.09	0.12	0.09	0.00	0.00	0.00		0.09
**2007**											
DSS (27), n	93	297	382	183	56	45	13	6	7	0	1082
DHF (26), n	2268	7105	11,099	11,005	4060	2105	854	359	169	29	39,053
DF (66), n	2009	6198	8197	5539	1805	940	454	172	124	8	25,446
Total dengue disease (26,27,66), n	4370	13,600	19,678	16,727	5921	3090	1321	537	300	37	65,581

Characteristic	Age group (years)										Total
	0–4	5–9	10–14	15–24	25–34	35–44	45–54	55–64	65+	Unknown age	
Deaths, n	13	10	10	17	5	3	0	1	0	0	59
Population, n	3,991,894	4,309,597	4,928,686	9,711,468	10,864,536	10,976,511	8,427,774	4,923,180	4,799,869		62,933,515
Incidence, /100,000	109.47	315.57	399.25	172.24	54.50	28.15	15.67	10.91	6.25		104.21
CFR	0.30	0.07	0.05	0.10	0.08	0.10	0.00	0.19	0.00		0.09
**2008**											
DSS (27), n	175	450	527	286	93	44	22	18	11	0	1626
DHF (26), n	3326	8069	12,907	15,140	6084	3727	1252	528	309	13	51,355
DF (66), n	2903	7804	11,046	8540	3249	1623	897	355	217	11	36,645
Total dengue disease (26,27,66), n	6404	16,323	24,480	23,966	9426	5394	2171	901	537	24	89,626
Deaths, n	12	16	21	9	7	2	0	3	0	0	70
Population, n	3,997,249	4,176,511	4,909,870	9,676,828	10,785,330	11,011,976	8,683,063	5,127,064	4,846,131		63,214,022
Incidence, /100,000	160.21	390.83	498.59	247.66	87.40	48.98	25.00	17.57	11.08		141.78
CFR	0.19	0.10	0.09	0.04	0.07	0.04	0.00	0.33	0.00		0.08
**2009**											
DSS (27), n	72	198	345	227	60	41	16	9	9	0	977
DHF (26), n	1784	4342	7545	8873	3937	2107	1099	484	292	17	30,480
DF (66), n	1808	4677	7207	6159	2566	1407	786	344	232	8	25,194
Total dengue disease (26,27,66), n	3664	9217	15,097	15,259	6563	3555	1901	837	533	25	56,651
Deaths, n	2	9	10	12	3	1	1	1	0	0	39
Population, n*	3,887,114	4,017,963	4,675,453	9,375,403	10,339,883	10,706,067	8,886,822	5,367,810	4,938,049	1,330,498	63,525,062
Incidence, /100,000	94.26	229.39	322.90	162.76	63.47	33.21	21.39	15.59	10.79		89.18
CFR	0.05	0.10	0.07	0.08	0.05	0.03	0.05	0.12	0.00		0.07

Characteristic	Age group (years)										Total
	0–4	5–9	10–14	15–24	25–34	35–44	45–54	55–64	65+	Unknown age	
**2010**											
DSS (27), n	236	745	907	648	238	116	81	37	20	0	3028
DHF (26), n	3665	8982	14,013	17,768	8239	4150	2350	1063	515	25	60,770
DF (66), n	3834	9644	13,598	13,667	6006	3171	1937	819	4595	14	53,149
Total dengue disease (26,27,66), n	7735	19,371	28,518	32,083	14,483	7437	4368	1919	994	39	116,947
Deaths, n	13	36	20	21	4	3	3	1	2	0	103
Population, n*	3,765,907	4,144,169	4,936,475	9,779,788	10,250,984	11,122,872	9,795,713	6,346,144	5,784,208		65,926,260
Incidence, /100,000	205.40	467.43	577.70	328.05	141.28	66.86	44.59	30.24	17.18		177.39
CFR	0.17	0.19	0.07	0.07	0.03	0.04	0.07	0.05	0.20		0.09
**2011**											
DSS (27), n	98	347	410	281	129	62	39	26	10	0	1402
DHF (26), n	2071	5170	8720	11,293	5509	3017	1628	782	447	2	38,639
DF (66), n	2225	5586	7429	7397	3309	1806	1088	569	350	0	29,759
Total dengue disease (26,27,66), n	4394	11,103	16,559	18,971	8947	4885	2755	1377	807	2	69,800
Deaths, n	7	13	9	5	8	3	2	0	0	0	47
Population, n**[†]	5,056,465	4,767,609	3,838,856	10,415,660	10,641,580	11,031,978	9,927,651	11,896,846			67,576,645
Incidence, /100,000	86.90	232.88	431.35	182.14	84.08	44.28	27.75	18.36			103.29
CFR	0.16	0.12	0.05	0.03	0.09	0.06	0.07	0.00	0.00		0.07

Empty cells: data not provided. CFR, case fatality rate; DF, dengue fever; DHF, dengue haemorrhagic fever; DSS, dengue shock syndrome.

*Data from the 2010 population and housing census, National Statistical Office [18]. ** Estimate

[†]Age bands for 2011 differ from other years (0–5, 6–10, 11–14, 15–24, 25–34, 35–44, 45–54, 55+) and do not match bands for disease numbers: consequently, some 2011 incidence figures are estimations.
