# Supplementary material for: Epidemiological Trends of Dengue Disease in Thailand (2000–2011): A Systematic Literature Review
Source: PLoS Negl Trop Dis. 2014 Nov 6;8(11):e3241. doi: 10.1371/journal.pntd.0003241 (PMC4222696; doi:10.1371/journal.pntd.0003241)
Supplement: Table S5 — Serotype distribution of dengue viruses in Thailand: national and/or regional studies. (PDF) [file pntd.0003241.s006.pdf]

Table S5. Serotype distribution of dengue viruses in Thailand: national and/or regional studies.

Year	Region	DENV-1 (%)	DENV-2 (%)	DENV-3 (%)	DENV-4 (%)	Cases tested (n)	Source of data
1998–2000	Kamphaeng Phet	23	35	41	1	108	Endy et al., 2002 [48]
2000	Lampang*	86	7	0/15	7	15	Anantapreecha et al., 2004 [50]
2000	Maharat Nakhon Ratchasima*	67	33	0	0	3	Anantapreecha et al., 2004 [50]
2000	Pathum Thani*	25	50	25	0	4	Anantapreecha et al., 2004 [50]
2000	Chareonkrug Pracharak*	50	40	10	0	10	Anantapreecha et al., 2004 [50]
2000	Ratchaburi*	42	4	52	2	238	Anantapreecha et al., 2004 [50]
2000	Hadyai*	95	0	5	0	22	Anantapreecha et al., 2004 [50]
2000[†]	Bangkok[†]	42	42	16	0	31	Sriprom et al., 2003 [38]
2000	Bangkok	47	27	17	10	31	Fried et al., 2010 [52]
2000–2001	Bangkok	100	0	0	0	64	Khawsak et al., 2003 [49]
2000–2001	Samut Prakan	0	50	0	50	2	Khawsak et al., 2003 [49]
2000–2001	Nakhon Sawan	12	12	41	35	17	Khawsak et al., 2003 [49]
2000–2001	Pathum Thani	0	70	0	30	10	Khawsak et al., 2003 [49]
2000–2001	Ratchaburi	0	19	31	50	23[‡]	Khawsak et al., 2003 [49]
2000–2001	Nakhon Pathom	25	25	33	17	33[‡]	Khawsak et al., 2003 [49]
2000–2001	Samut Songkhram	0	0	25	75	8	Khawsak et al., 2003 [49]
2000–2001	Ubon Ratchathani	0	44	11	44	10[‡]	Khawsak et al., 2003 [49]
2000–2001	Udon Thani	0	0	0[§]	100	7[‡]	Khawsak et al., 2003 [49]
2000–2001	Chian Rai	0	38	0	62	15[‡]	Khawsak et al., 2003 [49]
2000–2001	Phitsanulok	0	90	10	0	10	Khawsak et al., 2003 [49]
2000–2001	Ranong	0	83	0	17	6[‡]	Khawsak et al., 2003 [49]

Table S5. Serotype distribution of dengue viruses

Year	Region	DENV-1 (%)	DENV-2 (%)	DENV-3 (%)	DENV-4 (%)	Cases tested (n)	Source of data
2000–2001	Nakhon Si Thammarat	10	32	37	21	19‡	Khawsak et al., 2003 [49]
2000–2001	Yala	0	0	33	67	9‡	Khawsak et al., 2003 [49]
2001	Lampang*	57	23	19	1	150	Anantapreecha et al., 2004 [50]
2001	Maharat Nakhon Ratchasima*	47	39	9	5	176	Anantapreecha et al., 2004 [50]
2001	Pathum Thani*	41	41	13	5	143	Anantapreecha et al., 2004 [50]
2001	Chareonkrug Pracharak*	61	25	11	3	99	Anantapreecha et al., 2004 [50]
2001	Ratchaburi*	38	17	37	7	256	Anantapreecha et al., 2004 [50]
2001	Hadyai*	79	2	14	5	276	Anantapreecha et al., 2004 [50]
2001	Bangkok†	44	33	16	7	110	Sriprom et al., 2003 [38]
2001	Bangkok	42	42	13	2	45	Fried et al., 2010 [52]
2002	Lampang*	11	79	8	2	95	Anantapreecha et al., 2004 [50]
2002	Maharat Nakhon Ratchasima*	57	24	17	2	292	Anantapreecha et al., 2004 [50]
2002	Pathum Thani*	47	25	22	6	32	Anantapreecha et al., 2004 [50]
2002	Chareonkrug Pracharak*	65	25	5	5	65	Anantapreecha et al., 2004 [50]
2002	Ratchaburi*	12	64	7	7	170	Anantapreecha et al., 2004 [50]
2002	Hadyai*	44	17	17	22	77	Anantapreecha et al., 2004 [50]
2002	Chiangmai	48.7	35.9	0	15.4	39	Veeraseatakul et al., 2007 [51]
2002	Lamphun	33.3	66.7	0	0	6	Veeraseatakul et al., 2007 [51]
2002	Lampang	0	100	0	0	5	Veeraseatakul et al., 2007 [51]
2002	Mae Hong Son	0	0	0	0	0	Veeraseatakul et al., 2007 [51]
2002	Phrae	0	0	0	0	0	Veeraseatakul et al., 2007 [51]
2002	Northern region (total)	42.0	46.0	0	12.0	50	Veeraseatakul et al., 2007 [51]

Table S5. Serotype distribution of dengue viruses

Year	Region	DENV-1 (%)	DENV-2 (%)	DENV-3 (%)	DENV-4 (%)	Cases tested (n)	Source of data
2002[†]	Bangkok	46	44	8	2	50	Sriprom et al., 2003 [38]
2002	Bangkok	50	45	5	0	24	Fried et al., 2010 [52]
2002–2006	Northern region (total)	30.6	47.2	3.8	18.4	559	Veeraseatakul et al., 2007 [51]
2003	Chiangmai	33.3	54.8	4.7	7.2	42	Veeraseatakul et al., 2007 [51]
2003	Lamphun	0	100	0	0	2	Veeraseatakul et al., 2007 [51]
2003	Lampang	37.5	62.5	0	0	16	Veeraseatakul et al., 2007 [51]
2003	Mae Hong Son	0	100	0	0	2	Veeraseatakul et al., 2007 [51]
2003	Phrae	0	0	0	0	0	Veeraseatakul et al., 2007 [51]
2003	Northern region (total)	32.3	59.7	3.2	4.8	62	Veeraseatakul et al., 2007 [51]
2003[†¶]	Bangkok	42	23	34	1	29	Sriprom et al., 2003 [38]
2003	Central region	57.6	16.7	19.8	5.9		MoPH, 2003 [26]
2003	Northern region	73	8.3	12.9	5.9		MoPH, 2003 [26]
2003	Northeastern region	47.4	21.0	10.7	20.8		MoPH, 2003 [26]
2003	Southern region	57.5	16.4	15.9	10.2		MoPH, 2003 [26]
2004	Chiangmai	24.5	71.4	1.0	3.1	98	Veeraseatakul et al., 2007 [51]
2004	Lamphun	40.0	50.0	0	10.0	20	Veeraseatakul et al., 2007 [51]
2004	Lampang	17.2	79.3	3.5	0	29	Veeraseatakul et al., 2007 [51]
2004	Mae Hong Son	0	100	0	0	1	Veeraseatakul et al., 2007 [51]
2004	Phrae	0	0	0	0	0	Veeraseatakul et al., 2007 [51]
2004	Northern region (total)	25.0	70.3	1.4	3.3	148	Veeraseatakul et al., 2007 [51]
2004	Bangkok	40	10	2	48	50	Fried et al., 2010 [52]
2004	Central region	57.6	16.7	19.8	5.9		MoPH, 2004 [27]
2004	Northern region	73	8.3	12.9	5.9		MoPH, 2004 [27]
2004	Northeastern region	47.4	21.0	10.7	20.8		MoPH, 2004 [27]
2004	Southern region	57.5	16.4	15.9	10.2		MoPH, 2004 [27]
2004	Total	28.1	33.4	2.5	36.0		MoPH, 2004 [27]
2005	Chiangmai	24.0	52.0	5.3	18.7	75	Veeraseatakul et al., 2007 [51]

Table S5. Serotype distribution of dengue viruses

Year	Region	DENV-1 (%)	DENV-2 (%)	DENV-3 (%)	DENV-4 (%)	Cases tested (n)	Source of data
2005	Lamphun	8.8	17.6	11.8	61.8	34	Veeraseatakul et al., 2007 [51]
2005	Lampang	26.6	40.0	26.6	6.8	15	Veeraseatakul et al., 2007 [51]
2005	Mae Hong Son	8.3	75.1	8.3	8.3	12	Veeraseatakul et al., 2007 [51]
2005	Phrae	27.8	50.0	0	22.2	18	Veeraseatakul et al., 2007 [51]
2005	Northern region (total)	20.2	44.8	8.4	26.6	154	Veeraseatakul et al., 2007 [51]
2005	Bangkok	25	16	19	41	32	Fried et al., 2010 [52]
2005	Central region	39.6	9.8	4.5	46.1		MoPH, 2005 [28]
2005	Northern region	31.7	29.0	8.9	30.4		MoPH, 2005 [28]
2005	Northeastern region	39.2	13.5	4.0	43.3		MoPH, 2005 [28]
2005	Southern region	39.3	24.5	4.9	31.3		MoPH, 2005 [28]
2005	Total	37.5	18.4	5.7	38.4		MoPH, 2005 [28]
2006	Chiangmai	56.9	3.9	3.9	35.3	51	Veeraseatakul et al., 2007 [51]
2006	Lamphun	11.1	0	11.1	77.8	9	Veeraseatakul et al., 2007 [51]
2006	Lampang	71.4	0	14.3	14.3	7	Veeraseatakul et al., 2007 [51]
2006	Mae Hong Son	80.0	20.0	0	0	5	Veeraseatakul et al., 2007 [51]
2006	Phrae	31.5	38.4	0	30.1	73	Veeraseatakul et al., 2007 [51]
2006	Northern region (total)	42.8	21.3	2.8	33.1	145	Veeraseatakul et al., 2007 [51]
2006	Bangkok	40	2	35	23	44	Fried et al., 2010 [52]
2006	Central region	54.6	7.2	6.5	31.7		MoPH, 2006 [29]
2006	Northern region	54.2	7.1	6.3	32.4		MoPH, 2006 [29]
2006	Northeastern region	40.6	32.7	6.0	20.7		MoPH, 2006 [29]
2006	Southern region	42.9	30.4	7.1	19.6		MoPH, 2006 [29]
2006	Total	43.4	39.8	4.1	12.7	1766	MoPH, 2006 [29]
2007	Central region	54.8	11.9	22.9	10.4		MoPH, 2007 [30]
2007	Northern region	64.8	11.3	5.2	18.7		MoPH, 2007 [30]
2007	Northeastern region	69.6	13.2	9.7	7.5		MoPH, 2007 [30]
2007	Southern region	56.1	17.7	12.8	13.4		MoPH, 2007 [30]
2007	Total	60.9	12.5	14.9	11.7	2302	MoPH, 2007 [30]
2008	Central region	57.6	16.7	19.8	5.9		MoPH, 2008 [31]
2008	Northern region	73.0	8.3	12.9	5.9		MoPH, 2008 [31]

Table S5. Serotype distribution of dengue viruses

Year	Region	DENV-1 (%)	DENV-2 (%)	DENV-3 (%)	DENV-4 (%)	Cases tested (n)	Source of data
2008	Northeastern region	47.4	21.0	10.7	20.8		MoPH, 2008 [31]
2008	Southern region	57.5	16.4	15.9	10.2		MoPH, 2008 [31]
2008	Total	57.5	16.4	15.9	10.2	2987	MoPH, 2008 [31]
2009	Central region	39.5	23.5	32.4	4.6		MoPH, 2009 [32]
2009	Northern region	69.6	25.3	5.1	0		MoPH, 2009 [32]
2009	Northeastern region	60.0	40.0	0	0		MoPH, 2009 [32]
2009	Southern region	65.8	13.2	19.7	1.3		MoPH, 2009 [32]
2009	Total	47.6	28.8	20.2	3.4	555	MoPH, 2009 [32]
2010	Central region	25.8	45.0	15.1	6.2	225	MoPH, 2010 [33]
2010	Northern region	26.5	70.6	2.9	0	34	MoPH, 2010 [33]
2010	Northeastern region	41.7	41.7	16.7	0	12	MoPH, 2010 [33]
2010	Southern region	20.0	54.5	23.6	0	55	MoPH, 2010 [33]

DENV, dengue virus; DHF, dengue haemorrhagic fever; MoPH, Thai Ministry of Public Health.

*Data from one hospital in the province.
†DHF cases only.
‡Includes patients with mixed serotypes not included in stated percentages.
§Six patients had mixed DENV-3 and DENV-4 serotype.
¶Data for January–June.
